# Contributions from the silent majority dominate dengue virus transmission

**DOI:** 10.1371/journal.ppat.1006965

**Published:** 2018-05-03

**Authors:** Quirine A. ten Bosch, Hannah E. Clapham, Louis Lambrechts, Veasna Duong, Philippe Buchy, Benjamin M. Althouse, Alun L. Lloyd, Lance A. Waller, Amy C. Morrison, Uriel Kitron, Gonzalo M. Vazquez-Prokopec, Thomas W. Scott, T. Alex Perkins

**Affiliations:** 1 Department of Biological Sciences, Eck Institute for Global Health, University of Notre Dame, Notre Dame, IN, United States; 2 Department of Epidemiology, Johns Hopkins School of Public Health, Baltimore, MD, United States; 3 Insect-Virus Interactions Group, Department of Genomes and Genetics, Institut Pasteur, Paris, France; 4 Centre National de la Recherche Scientifique, Unité Mixte de Recherche 2000, Paris, France; 5 Virology Unit, Institut Pasteur du Cambodge, Institut Pasteur International Network, Phnom Penh, Cambodia; 6 GlaxoSmithKline, Vaccines R&D, Singapore; 7 Institute for Disease Modeling, Bellevue, WA, United States; 8 Information School, University of Washington, Seattle, WA, United States; 9 Department of Biology, New Mexico State University, Las Cruces, NM, United States; 10 Department of Mathematics, Biomathematics Graduate Program and Center for Quantitative Sciences in Biomedicine, North Carolina State University, Raleigh, NC, United States; 11 Department of Biostatistics and Bioinformatics, Rollins School of Public Health, Emory University, Atlanta, GA, United States; 12 Department of Entomology and Nematology, University of California, Davis, CA, United States; 13 Department of Environmental Sciences, Emory University, Atlanta, GA, United States; Imperial College London, UNITED KINGDOM

## Abstract

Despite estimates that, each year, as many as 300 million dengue virus (DENV) infections result in either no perceptible symptoms (asymptomatic) or symptoms that are sufficiently mild to go undetected by surveillance systems (inapparent), it has been assumed that these infections contribute little to onward transmission. However, recent blood-feeding experiments with *Aedes aegypti* mosquitoes showed that people with asymptomatic and pre-symptomatic DENV infections are capable of infecting mosquitoes. To place those findings into context, we used models of within-host viral dynamics and human demographic projections to (1) quantify the net infectiousness of individuals across the spectrum of DENV infection severity and (2) estimate the fraction of transmission attributable to people with different severities of disease. Our results indicate that net infectiousness of people with asymptomatic infections is 80% (median) that of people with apparent or inapparent symptomatic infections (95% credible interval (CI): 0–146%). Due to their numerical prominence in the infectious reservoir, clinically inapparent infections in total could account for 84% (CI: 82–86%) of DENV transmission. Of infections that ultimately result in any level of symptoms, we estimate that 24% (95% CI: 0–79%) of onward transmission results from mosquitoes biting individuals during the pre-symptomatic phase of their infection. Only 1% (95% CI: 0.8–1.1%) of DENV transmission is attributable to people with clinically detected infections after they have developed symptoms. These findings emphasize the need to (1) reorient current practices for outbreak response to adoption of pre-emptive strategies that account for contributions of undetected infections and (2) apply methodologies that account for undetected infections in surveillance programs, when assessing intervention impact, and when modeling mosquito-borne virus transmission.

## Author summary

Most dengue virus infections result in either no perceptible symptoms or symptoms that are so mild that they go undetected by surveillance systems. It is unclear how much these infections contribute to the overall transmission and burden of dengue. At an individual level, we show that people with asymptomatic infections are approximately 80% as infectious to mosquitoes as their symptomatic counterparts. At a population level, we show that approximately 88% of infections result from people who display no apparent symptoms at the time of transmission. These results suggest that individuals undetected by surveillance systems may be the primary reservoir of dengue virus transmission and that policy for dengue control and prevention must be revised accordingly.

## Introduction

Though often assumed benign, it is increasingly recognized that for many pathogens, clinically inapparent infections can represent a sizeable portion of the infectious reservoir [1–3] and contribute substantially to pathogen transmission [4]. Understanding the contribution to transmission from people with inapparent infections is fundamental for inferring drivers of transmission [3], estimating the timing and scope of outbreaks [5], planning and monitoring control efforts [6], and assessing the feasibility of pathogen elimination [1,6].

Of the 390 million dengue virus (DENV) infections that occur globally each year, an estimated 300 million do not result in symptoms severe enough for a person to seek treatment [7,8], meaning that they likely go undetected by most surveillance systems. The four closely related DENV1-4 serotypes are transmitted predominantly by *Aedes aegypti* mosquitoes [9], and infection with one serotype is believed to be followed by short-term, heterologous cross-immunity and life-long homologous immunity [10]. Based on observed positive correlations between DENV viremia and disease severity [11–14], it has been assumed that the 300 million annual clinically inapparent infections contribute little to onward transmission because their viremia levels are too low to efficiently infect mosquitoes. On the other hand, high sero-conversion rates coinciding with few reported cases in some areas suggest that inapparent infections may contribute appreciably to silent DENV transmission [15]. Furthermore, recent blood-feeding experiments with *Ae*. *aegypti* mosquitoes demonstrated that people with asymptomatic and pre-symptomatic DENV infections are indeed capable of infecting mosquitoes [16].

Although these indications of a possible role of inapparent infections in DENV transmission have become more evident, the proportion of overall transmission for which they are responsible is unknown. This potentially significant unknown has important implications for policy given the difficulty of identifying people with inapparent infections, which may be a critical technical limitation in the event that they contribute appreciably to transmission.

**Fig 1:**
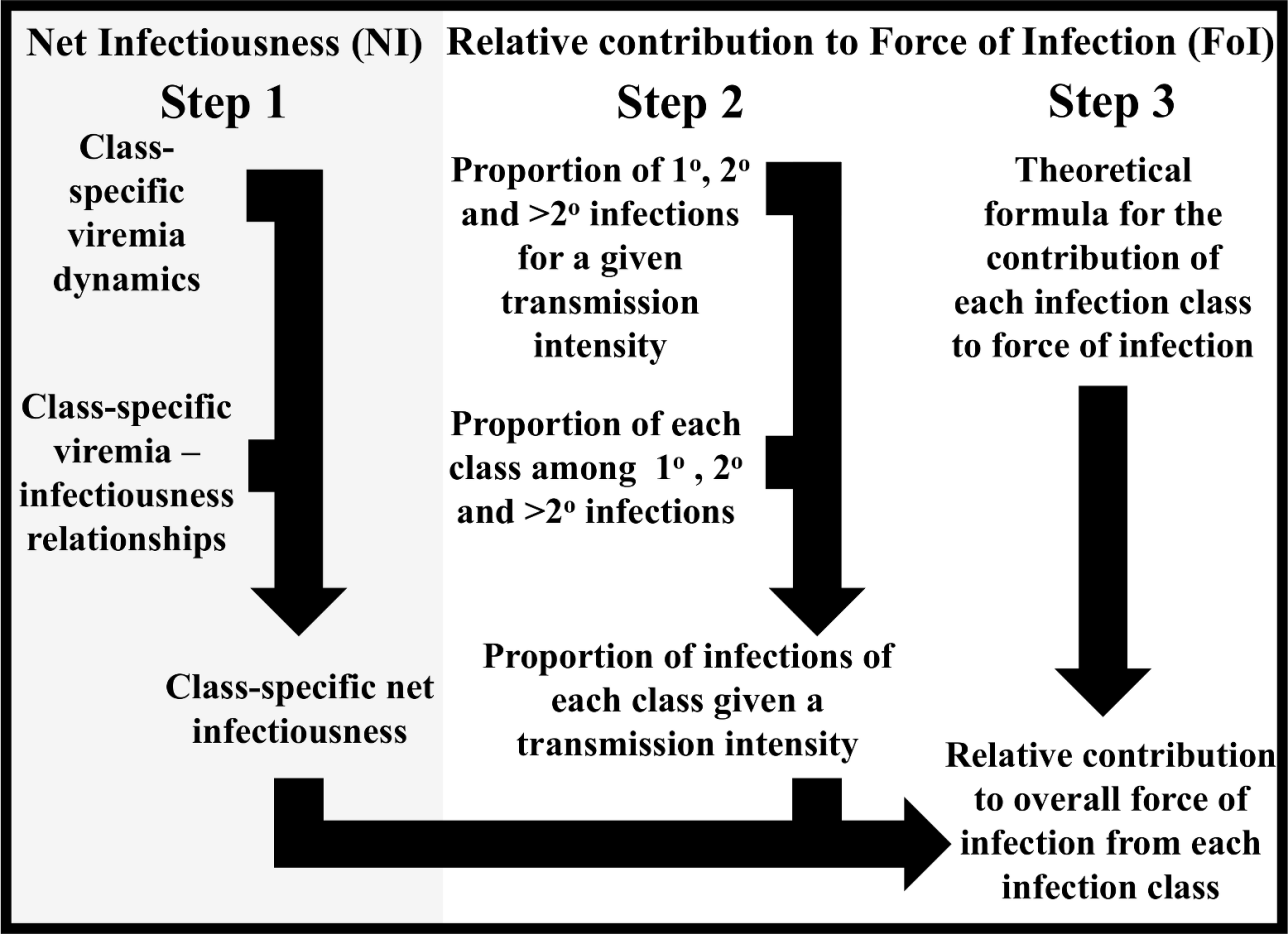
Flowchart of methodological framework. Our overall approach consisted of three steps, depicted in the three columns. The products of all three combine to yield our estimate of the relative contribution to overall force of infection from each infection class. Likewise, uncertainty in each component of the analysis is propagated into other components of the analysis through repeated Monte Carlo sampling, with Monte Carlo samples from one portion of the analysis flowing to others according to the arrows and providing the basis for credible intervals reported in the *Results* section. Details of how the sub-steps indicated in this figure were performed are described in subsections of the *Materials and Methods* section.

We addressed this question by estimating the net infectiousness (NI) of DENV-infected individuals with different clinical manifestations, including asymptomatic infections, and quantifying the relative contributions of these clinically distinct classes to the overall force of infection (FoI) of DENV. Our approach involved three distinct steps (Fig 1). First, we estimated the net infectiousness of different classes of DENV infections by combining class-specific estimates of DENV viremia trajectories and class-specific estimates of the relationship between DENV viremia and infectiousness to mosquitoes. Second, we estimated the proportion of infections of each class by first quantifying the proportion of primary, secondary, and post-secondary DENV infections in a population with a given level of transmission intensity. We then translated those proportions of primary, secondary, and post-secondary infections into proportions of each infection class based on estimates of those relationships from the literature. Third, we used a novel formulation of DENV force of infection to combine estimates of individual-level net infectiousness of each infection class and estimates of the proportion of each infection class in a hypothetical population to estimate the proportional contribution of each infection class to overall DENV force of infection.

## Results

### Definitions

**Fig 2:**
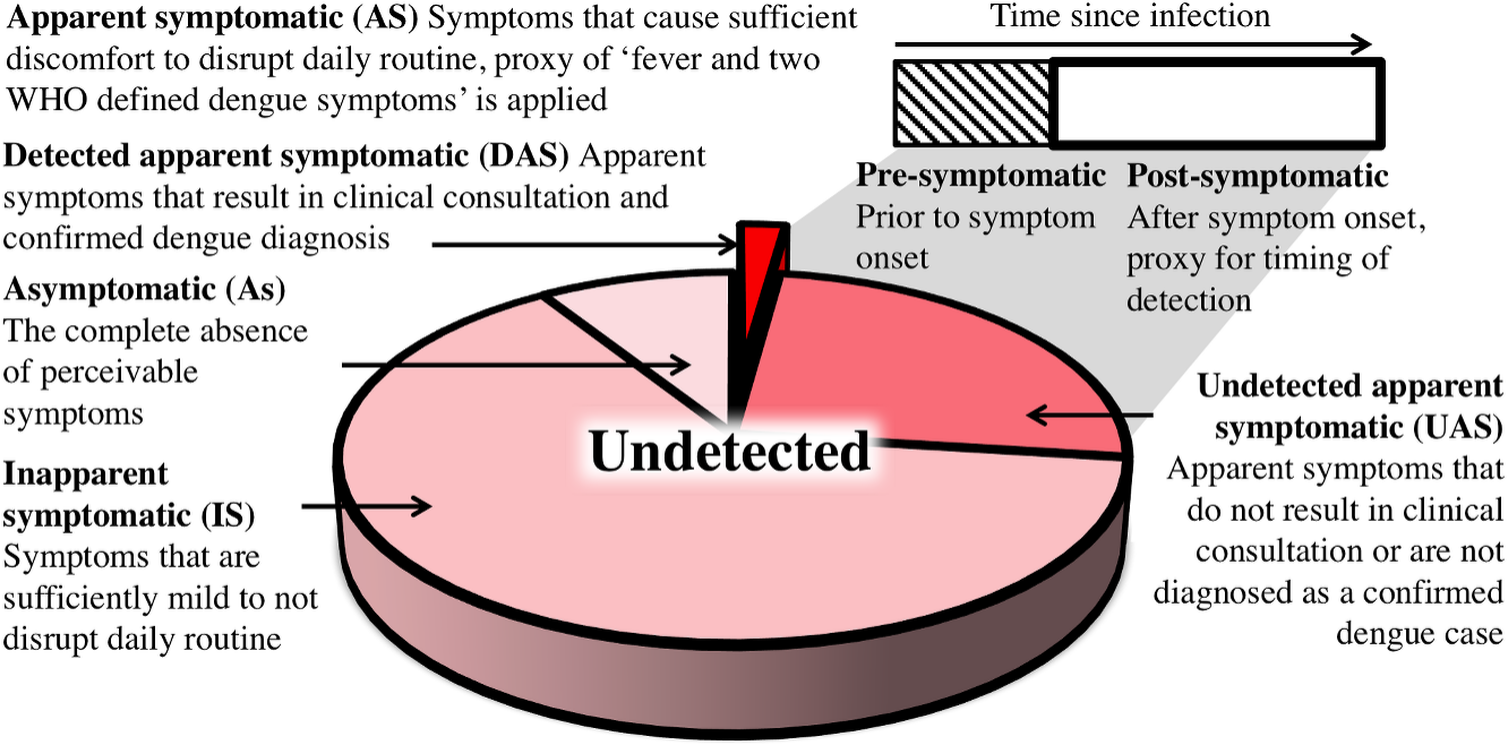
Definitions of DENV infection classes.

We distinguished four classes of infections (Fig 2). We referred to the first class as “asymptomatic” (As), which we defined as people having absolutely no perceptible symptoms at any point during their infection [16]. The remaining people with symptomatic (S) infections were divided into: (1) inapparent symptomatic (IS), people whose symptoms are sufficiently mild to not disrupt their daily routine and thus do not prompt healthcare seeking [7,16]; and (2) apparent symptomatic (AS) individuals, whose clinical presentation does disrupt their daily routine according to the WHO definition of “at least fever and two dengue symptoms” [17]. Detected apparent symptomatic individuals (DAS) are identified by health surveillance systems if they seek clinical consultation and are diagnosed as a confirmed dengue case. Others remain undetected (UAS) (Fig 2).

### Net infectiousness (NI)

We quantified differences in the net infectiousness to mosquitoes of individuals with As, IS, and AS infections. We modeled viral transmission dynamics of each of these infection types by using a mechanistic model of the within-host dynamics of DENV fitted to plasma viral titers over time for patients with S infections [18] and adjusted these trajectories using an empirically informed distribution of correction ratios to model trajectories of As infections [16]. Secondary (2°) infections were parameterized to exhibit faster cell entry and accelerated clearance of viral particles than primary (1°) infections, consistent with theory for antibody-dependent enhancement and increased activation of the immune system [18]. This resulted in a shorter duration of detectable, and potentially infectious, viremia [19–21]. Post-2° infections were excluded from consideration for our primary analysis due to a combination of low risk of apparent disease and high rates of cross-reactive antibodies for this group [22]. Nonetheless, because there is no conclusive evidence about viremia of post-2° infections [22], we performed a sensitivity analysis in which an upper limit for post-2° viremia was assumed similar to that of 2° infections given that the dynamics of both are limited by varying degrees of immunity (S1 Fig). Next, we applied logistic regression models [16] to infer infectiousness to mosquitoes from human viral titers (S1 and S4 Tables, S2 Text) (see *Materials and Methods* for details).

**Fig 3:**
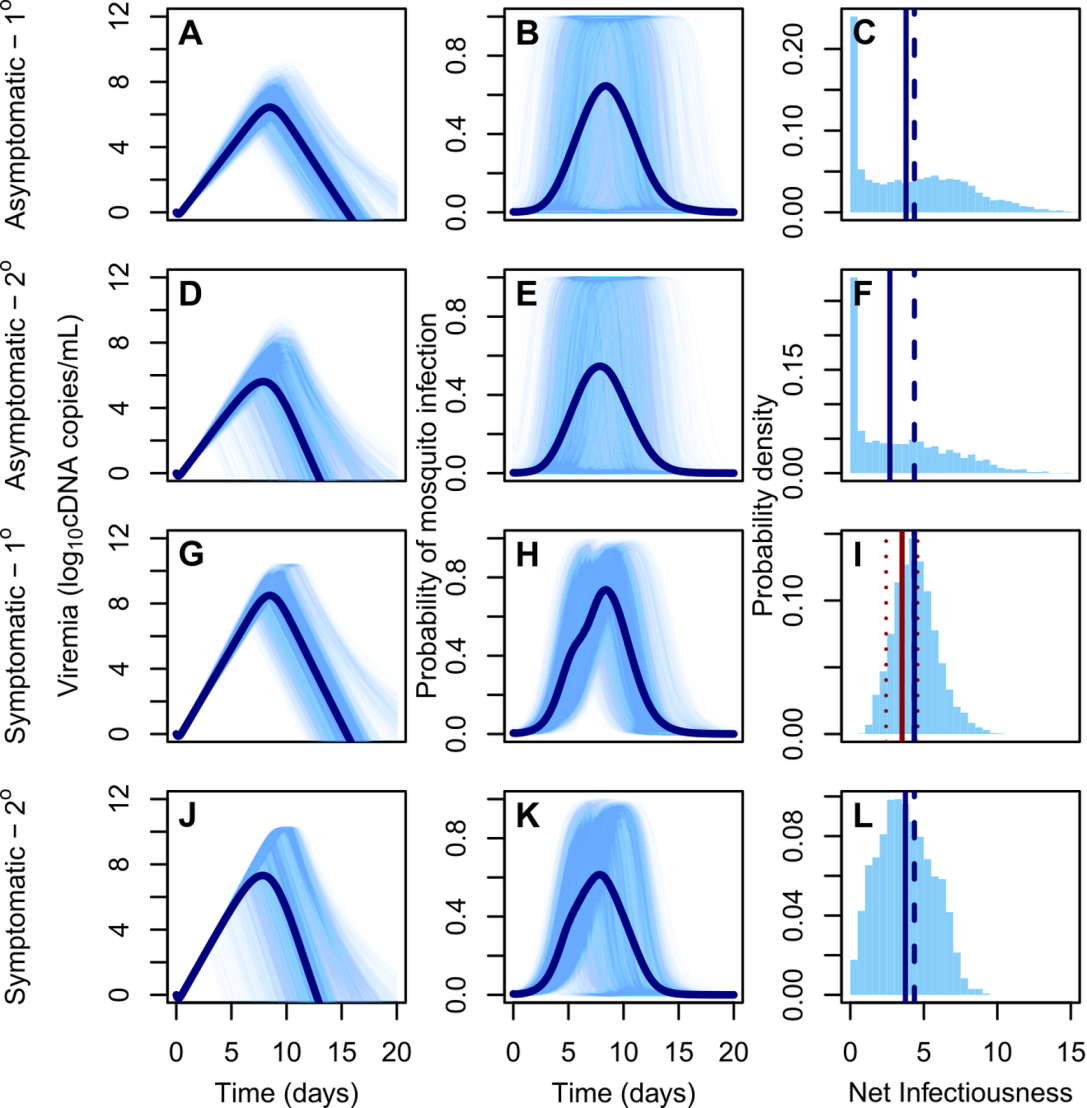
DENV viremia and infectiousness trajectories by infection class. (A,D,G,J): DENV viremia since time of infection for different infection classes and pre-exposure histories (1°: primary infection; 2°: secondary infection). Lighter lines denote 3,000 replicates and dark lines means. (B,E,H,K): Infectiousness of humans to mosquitoes over time. (C,F,I,L): Probability density of net infectiousness as defined in Eq (4) based on curves from the middle column. The solid blue line denotes the median and the dashed line denotes the median for the reference group (1° symptomatic). The solid and dashed red lines denote the mean and 95% confidence interval of the net infectiousness of 1° symptomatic infections as measured empirically [63].

The median NI to *Ae*. *aegypti* of As infections was lower than that of S infections, but of similar magnitude (1°: 87%, 95% CI: 0–151%; 2°: 74%, 95% CI: 0–137%). The median NI of 1° infections was greater than that of corresponding 2° infections (As: 140%, 95% CI: 111–275%; S: 120%, 95% CI: 100–235%) (Fig 3). Approximately one quarter of the NI of S infections occurred before symptom onset (1°: 21%, 95% CI: 1–56%, 2°: 27%, 95% CI: 0–97%). By calculating the probability that a random draw from the NI distribution of one infection class was lower than a random draw from another class (Pr), we confirmed that As infections are more likely to be less infectious than S infections (1°: Pr = 0.55; 2°: Pr = 0.58) and 2° infections are more likely to be less infectious than 1° infections (As: Pr = 0.56; S: Pr = 0.62) (S2 Table). There was wide variability in the NI of As infections, however, with both two-fold lesser or greater infectiousness compared to S infections appearing probable (lesser, 1°: 0.38, 2°: 0.42; greater, 1°: 0.16, 2°: 0.20). Overall, 1° As infections were not significantly less infectious than 2° S infections (Wilcoxon rank sum test, *p* = 0.97).

### Relative contributions to force of infection (FoI)

Next, we estimated the proportion of each infection class in a hypothetical population and derived each class’ relative contribution to FoI, the rate at which susceptible people become infected. We assumed an equal probability of being bitten by *Ae*. *aegypti* across infection classes. To quantify the proportion of people with As, IS, UAS, and DAS infections (and thus the pool of individuals who could potentially give rise to new infections among susceptibles), we used estimates from a recent meta-analysis on published (As+IS):AS ratios for primary and secondary infections (1°: 82%, 95% CI: 81–84; 2°: 76%, 95% CI: 74–78) [23]. Post-2° infections were assessed separately, using studies compiled by [7]. A beta-binomial model of these proportions was supported over a binomial model (deviance information criterion (DIC) binomial: 163, beta-binomial: 34). We estimated the (As+IS):AS ratio among post-2° infections to be higher than that of 1° and 2° infections (86%, 95% CI: 63–97). For all pre-exposure histories we adopted an As:(IS+AS) ratio of 9.2% (95% CI: 4.4–14.0%), which reflects the proportion of asymptomatic infections detected among cluster participants in [16] and a detection rate of AS infections of 8% (95% CI: 5–15%) [24] (see sensitivity analysis for an assessment of the full range of As:IS:AS ratios).

**Fig 4:**
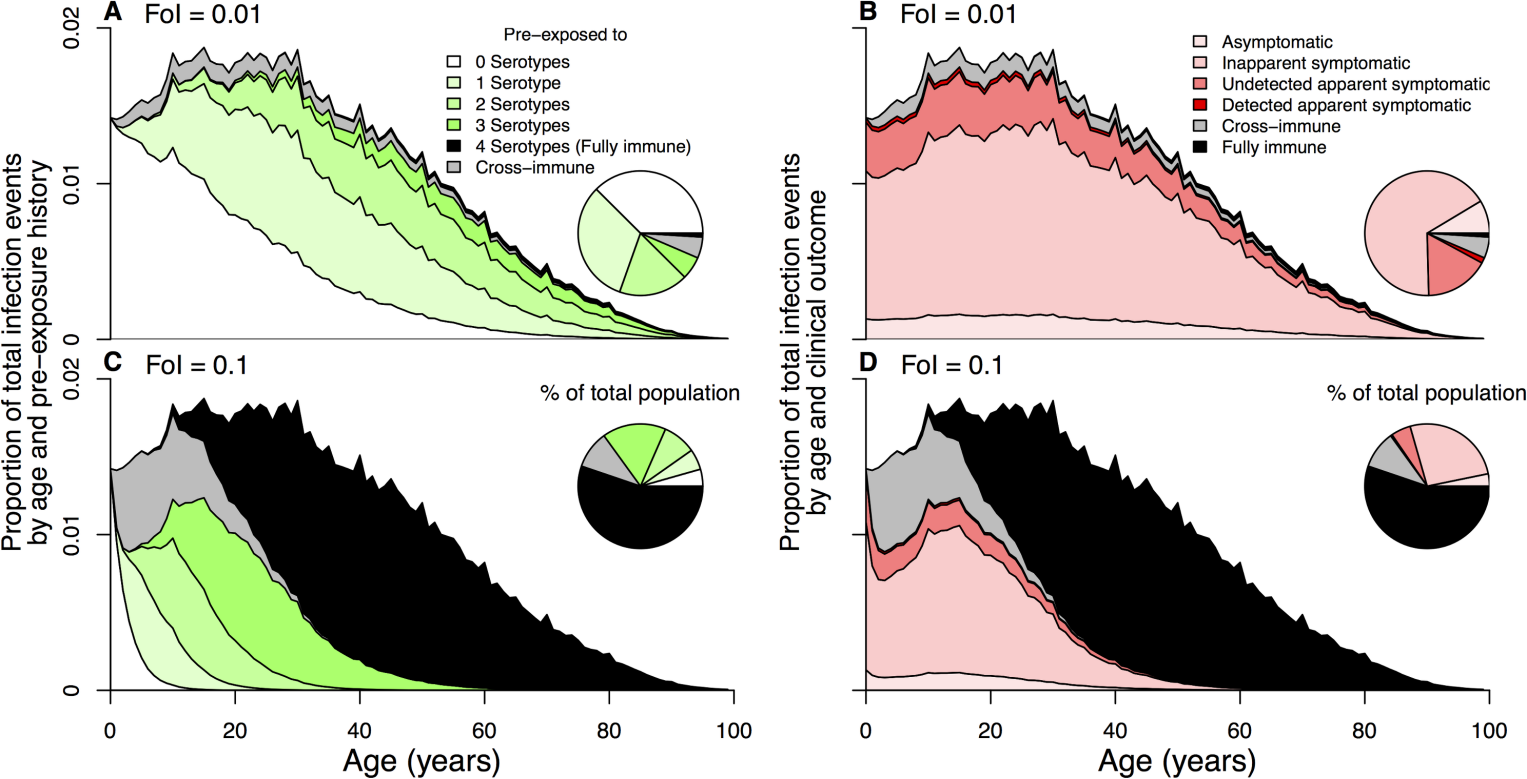
Pre-exposure history (A and C) and infection class (B and D) stratification by age and for FoI values of 0.01 (top) and 0.1 (bottom). An individuals’ susceptibility to infection and clinical outcome depend on pre-exposure history. Serohistory by age (A and C) is estimated using a system of ordinary differential equations with state variables denoting the proportion of the population pre-exposed to 0–4 serotypes. Transition to pre-exposure state *i* occurs at rate *i*FoI. Individuals entering a new pre-exposure state have temporary heterologous immunity (gray) to all serotypes before later becoming susceptible again to each serotype to which they do not have a history of exposure. After four infections with four different serotypes, individuals are assumed fully immune (black) to all serotypes.

The proportion of individuals with a given pre-exposure history (e.g., no previous DENV exposure, prior exposure to one serotype), or with temporary heterotypic immunity or permanent homotypic immunity, depends on the age distribution and the history of local transmission intensity [25]. We considered scenarios for our hypothetical populations with demographic characteristics of Brazil and Thailand, respectively, and simulated pre-exposure history by age across values of time-averaged FoI [26] (Figs 4 and S2). Combining the aforementioned inapparent ratios with realistic demographic scenarios in a location with a given overall, time-averaged FoI, we derived formulas

$$\begin{array}{l} {\text{FoI}}_{\text{As}}=bma\frac{\sum_{i=1}^{p}NI_{\text{As}i}\sigma R_{\text{As}i}}{\frac{g}{a}+\sum_{i=1}^{p}NI_{\text{As}i}\sigma R_{\text{As}i}}e^{-gn},\\ {\text{FoI}}_{\text{As+IS+UAS+DAS}}=bma\frac{\sum_{i=1}^{p}NI_{\text{As}i}\sigma R_{\text{As}i}+\sum_{i=1}^{p}NI_{\text{IS}i}\sigma R_{\text{IS}i}+\sum_{i=1}^{p}NI_{\text{UAS}i}\sigma R_{\text{UAS}i}+\sum_{i=1}^{p}NI_{\text{DAS}i}\sigma R_{\text{DAS}i}}{\frac{g}{a}+\sum_{i=1}^{p}NI_{\text{As}i}\sigma R_{\text{As}i}+\sum_{i=1}^{p}NI_{\text{IS}i}\sigma R_{\text{IS}i}+\sum_{i=1}^{p}NI_{\text{UAS}i}\sigma R_{\text{UAS}i}+\sum_{i=1}^{p}NI_{\text{DAS}i}\sigma R_{\text{DAS}i}}e^{-gn}, \end{array}$$ (1)

for the FoI attributable to As infections that can be divided by the overall FoI as FoI_As_ / FoI_As+IS+UAS+DAS_ to quantify the proportional contribution to FoI from the As class. The same approach can be applied to derive the proportional contribution to FoI of any other infection class. The parameters in Eq (1) are defined in S1 Table and account for different aspects of local dengue epidemiology and the aforementioned descriptions of differential net infectiousness and prevalence of As, IS, and AS infections with distinct pre-exposure histories (see *Materials and Methods* for additional details).

**Fig 5:**
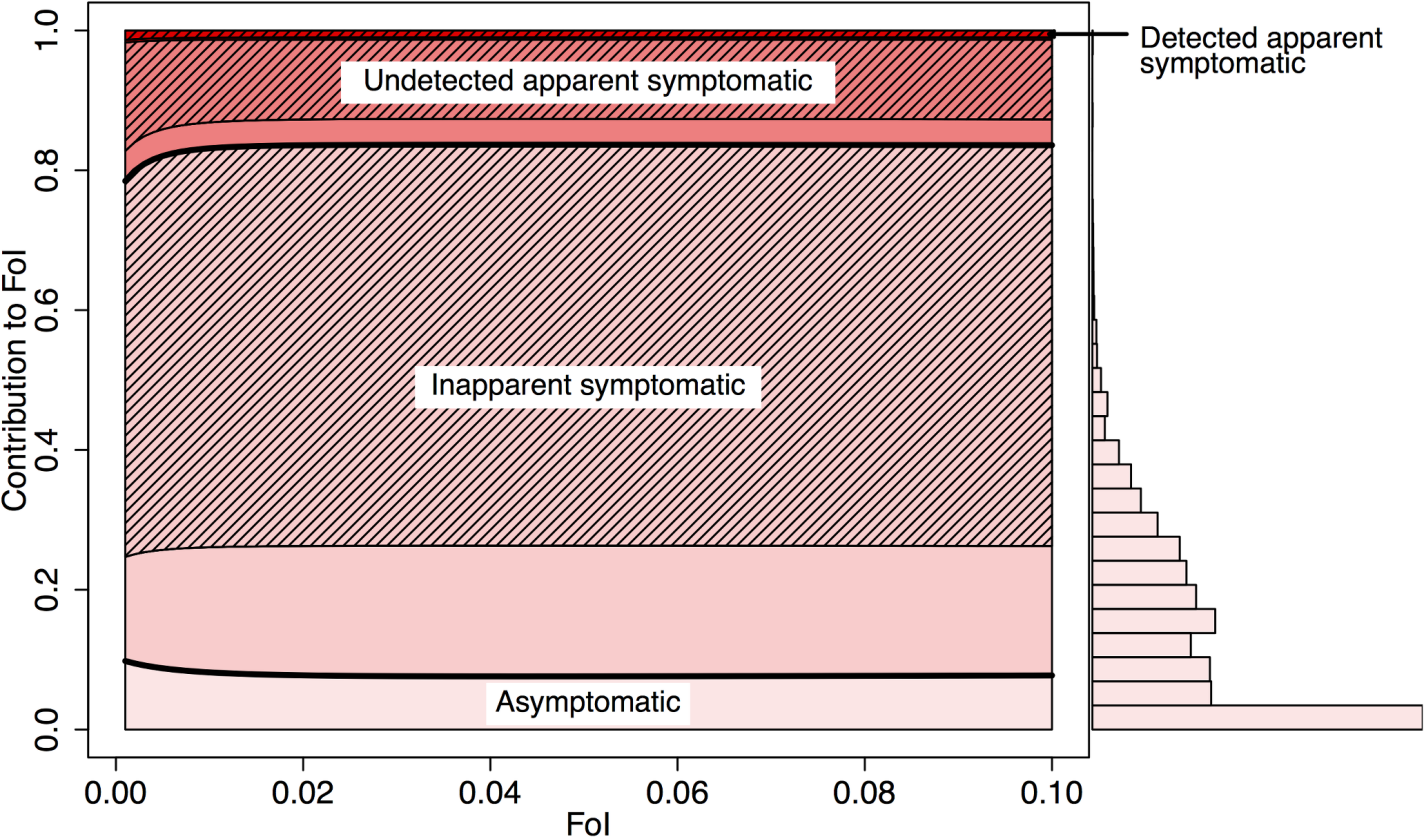
Mean contribution of each infection class to total force of infection (FoI). The contribution to the total FoI of an infection class is derived from the ratio of FoI attributable to a given class and total FoI, as in Eq (1). The respective net infectiousness is derived from the 3,000 random samples displayed in Fig 3. The infections are further distributed according to the estimated proportion of net infectiousness to occur before and after symptom onset (pre-symptomatic (Eq (5)) and post-symptomatic (hatched lines) (Eq (6)). The histogram shows the distribution of FoI contributions by asymptomatic infections at FoI = 0.1, accounting for parameter uncertainty.

Based on our metric of relative FoI, we estimated that 88% (95% CI: 77–92%) of human DENV infections are attributable to individuals that do not present with apparent symptoms at the time when they are bitten by a susceptible mosquito (i.e., As, IS, and pre-symptomatic AS) (Figs 5 and S3 for Thailand). We estimated that As and IS infections could together be responsible for causing 84% (95% CI: 82–86%) of all human DENV infections, reflecting a near one-to-one relationship with their representation in the population. Of the remaining 16% (95% CI: 14–18%) of infections, 76% (95% CI: 21–100%) are attributable to bites by mosquitoes on people whose infection eventually becomes apparent, and thus potentially detectable, after onset of symptoms. At a detection rate of 8% [24], an estimated 1.3% (95% CI: 1.1–1.4%) of total infections result from infected individuals after they are detected by surveillance systems (0.8%, 95% CI: 0.7–0.9%; 2.5%, 95% CI: 2.1–2.7% at detection rates of 5% and 15% [24], respectively).

### Sensitivity analysis

Data on viremia and infectiousness in asymptomatic, pre-symptomatic, and post-2° infections are sparse [16], resulting in substantial uncertainty around our estimates. We performed a variance-based sensitivity analysis [27] to identify the primary sources of uncertainty for estimating net infectiousness and the proportion of net infectiousness occurring prior to symptom onset. Limited data on the viremia-to-infectiousness relationship of asymptomatic infections drove the majority of uncertainty in estimates of their net infectiousness, whereas uncertainty in time until symptom onset was responsible for the majority of uncertainty in estimates of the proportion of net infectiousness prior to symptom onset (S4 Fig).

The estimated contributions to FoI by each infection class were robust across different transmission settings (Fig 5) and in settings where DENV is newly emerging (S5 Fig), but not when allowing for contributions to transmission from post-2° infections (S1 Fig). Under the assumption that the net infectiousness of post-2° infections is equivalent to that of 2° infections, we estimated the contribution of inapparent infections to be up to 11% (95% CI: 10–13%) higher than if post-2° infections had not been assumed to contribute to transmission (S1 Fig). This increase resulted from the relatively high proportion of IS infections among post-2° infections, who made up a larger proportion of the infectious reservoir in more intense transmission settings. Under the assumption that IS infections are more similar in their infectiousness to As than to AS infections, the estimated contribution of inapparent infections was reduced from 84% to 73% (95% CI: 0–91%), reflecting a lower bound on this assumption. The impact of accounting for the differential viral trajectories of severe dengue cases [18] was minor due to their small numerical prominence [28], but their inclusion did increase the contribution of post-symptomatic DAS infections from 1.0% (95% CI: 0.8–1.1%) to 2.1% (95% CI: 0.8–3.6).

**Fig 6:**
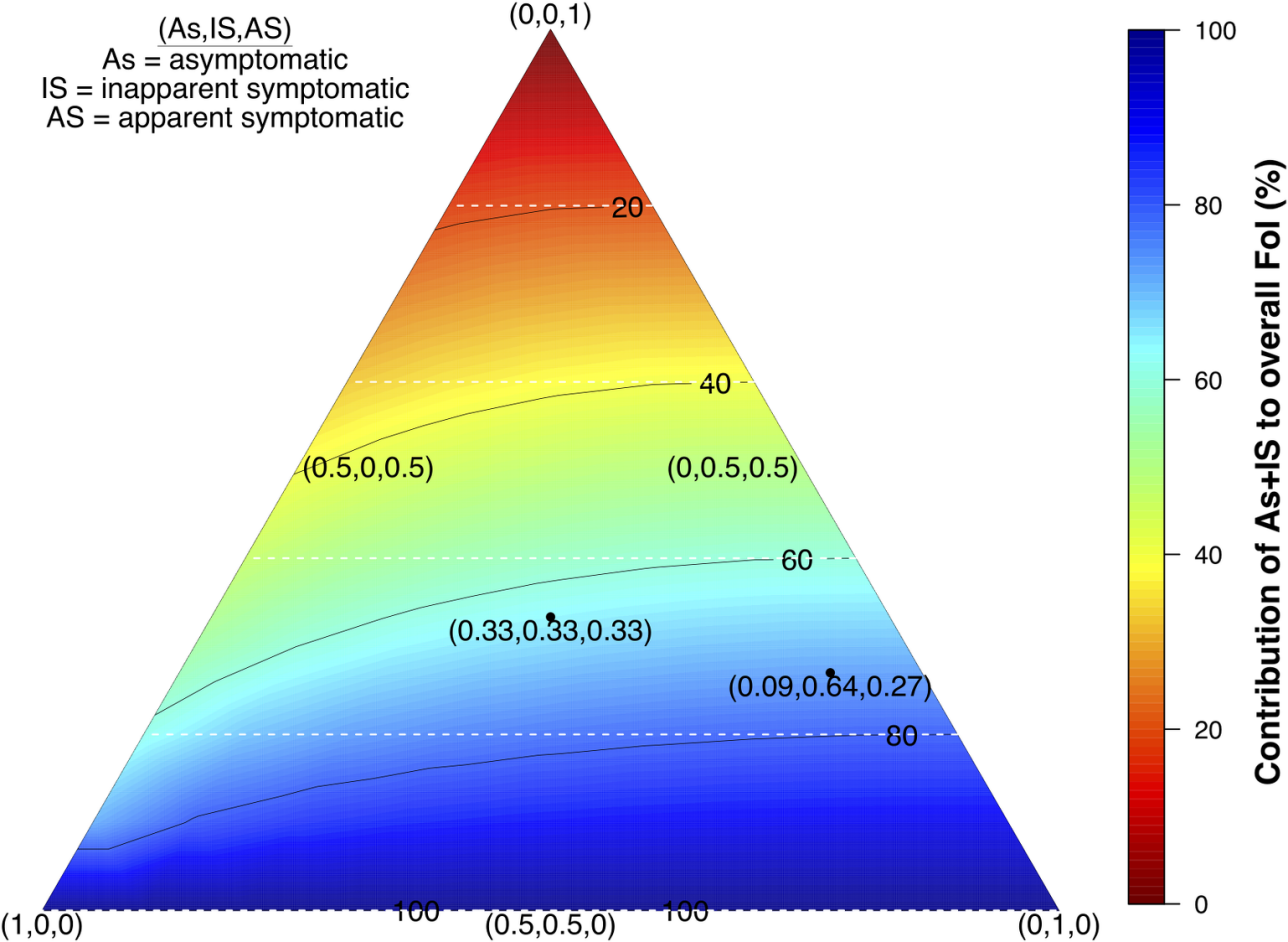
Contribution to the total force of infection (FoI) of inapparent infections (As+IS) for different As:IS:AS ratios (FoI = 0.05). Darker blue colors indicate a larger contribution to the FoI by As+IS infections in the population. The coordinates reflect the proportions per infection class with (0.09, 0.64,0.27) reflecting a default based on [16]. The white lines indicate the expected proportion of inapparent contribution if all types of infections had an equal net infectiousness.

As:IS:UAS:DAS ratios can fluctuate in space and time; for instance, as a result of serotype differences in virulence [7,23]. The contribution of inapparent (As+IS) infections across the spectrum of As:IS:AS ratios is driven by the ratio of As to IS infections, with the total contribution of As+IS infections generally being similar to their numerical prominence in the infected population (Fig 6). Although variability in As:IS:AS ratios likely does drive some variability in the contribution of As+IS infections, our analysis suggests that their contribution is unlikely to be less than 81% of their numerical prominence (Fig 6).

## Discussion

Combined with recent empirical findings [16,18], our modeling analysis suggests that inapparent infections contribute appreciably to DENV transmission and its disease burden via infection of others who develop clinical symptoms. In large part, our results stem from the large numerical prominence of As and IS DENV infections and the finding that differences in the viremia profiles of As and S infections are insufficient to result in considerable differences in net infectiousness. Moreover, our finding that approximately one quarter of an individual’s infectiousness occurs prior to symptom onset supports the hypothesis that a large proportion of human-to-mosquito transmission is silent; i.e., it happens when there is no detectable illness [15,16].

The substantial role that inapparent infections play during dengue epidemics may result in more rapid transmission and geographic spread [3] and, as a result, more widespread transmission prior to case-driven outbreak detection and onset of control efforts [29]. The considerable potential for pre-symptomatic transmission further impedes the use of case data to predict whether an outbreak will occur and, if it does, what its final size will be [30]. Despite increasing recognition of the importance of silent transmission to the epidemiology of a variety of pathogens, it is rarely taken into account in dengue models [31–34].

Our results also highlight the potential significance of a key uncertainty in projections of the population-level impact of the recently licensed Dengvaxia vaccine, which is thought to protect some vaccine recipients against apparent disease but not infection [35]. Although spared from disease, our results imply that breakthrough infections in these individuals could appreciably contribute to transmission and, therefore, limit the indirect effects of vaccination [31,36]. If individuals with asymptomatic or inapparent infections are as infectious as their apparent symptomatic counterparts, positive indirect effects of vaccine implementation in highly endemic settings would be reduced [31,32]. Conversely, adverse effects of vaccination in low-transmission settings, due to higher rates of severe disease in DENV-naïve vaccine recipients [37], would be offset to some degree if inapparent infections are important contributors to transmission given that this would increase the projected indirect effects of vaccination in those settings [31,32].

The contribution of silent infections to transmission helps explain the difficulty of carrying out control efforts in response to reported cases, particularly in intense transmission settings [29]. That approach could be further impeded by substantial inter-individual variability in pre-symptomatic and asymptomatic infectiousness [29]. If some people with silent infections are substantially more infectious than those with apparent infections, a significant portion of DENV transmission clusters would not be detected or contained by traditional surveillance efforts. Additional data are required to determine to what extent the observed variability in infectiousness results from uncertainty in the data or variability among individuals [33,38]. Improving strategies with enhanced potential to prevent infections, silent or otherwise, requires a deeper understanding of the spatial and temporal scales of transmission [39].

Our results underscore the need to resolve substantial uncertainties about within-host DENV dynamics, in particular with respect to viremia and infectiousness of asymptomatic, pre-symptomatic, and post-2° infections. An improved understanding of differences in viremia profiles between 1° and 2° infections, and the mechanisms underlying those processes requires more detailed immunological and viremia titer data, including quantifications of infectious particles, particularly from earlier stages of the infection [38,40]. Similarly, a greater understanding of the factors underlying differences in viral trajectories [38,41,42], infectiousness [41–43], and disease rates [23] between serotypes is needed to gain better, more context-specific estimates of the contributions of silent transmission to DENV transmission. Further, uncertainty about the net infectiousness of post-2° infections limits understanding of dengue dynamics overall [44] and the contribution of silent transmission in particular. Acquiring this kind of empirical data is complicated by the difficulty of identifying people in these classes when they are viremic and the absence of diagnostic tools that reliably distinguish 2° from post-2° infections [45]. Moreover, people with symptomatic infections may experience impaired mobility [46] or otherwise modified interactions with mosquitoes, the impacts of which on net transmission are presently not well understood. Resolving these uncertainties and identifying effective strategies for mitigating the contributions of all infections, apparent or not, to DENV transmission will require comprehensive studies that combine field work and modeling to address the coupled nature of multiple transmission heterogeneities [33] (see S1 Text for a comprehensive discussion of additional limitations and research needs).

## Conclusion

Heterogeneous infectiousness coupled with heterogeneity in disease symptoms is common across a wide range of pathogens [47]. A more thorough, quantitative understanding of the factors underlying these heterogeneities and their role in pathogen transmission is critical for a more complete understanding of pathogen dynamics and, in turn, enhanced disease prevention [48]. Our results advance understanding of these heterogeneities for dengue, showing that they are significant at both individual and population levels. In particular, our findings suggest that failing to acknowledge these coupled heterogeneities may significantly handicap understanding of DENV transmission dynamics, the effectiveness of dengue outbreak response efforts, and the evaluation of novel interventions for dengue prevention and control. These results show that additional emphasis on model-based approaches that bridge individual-level infection and population-level transmission processes have the potential to provide valuable insights for infectious disease prevention and control and for the identification of high-priority needs for future data collection.

## Materials and methods

### Class-specific viremia dynamics

We modeled viremia trajectories (log_10_ cDNA copies/mL of plasma) using a model [18] of virus and immune dynamics with four state variables—uninfected target cells (*x*), infected targets cells (*y*), free viral particles (*v*), and a clearing immune response (*z*)—according to

$$\begin{array}{l} \frac{dx}{dt}=A-\gamma x-\beta xv\\ \frac{dy}{dt}=\beta xv-\delta y-\alpha zy\\ \frac{dv}{dt}=\omega y-\kappa v\\ \frac{dz}{dt}=\eta yz. \end{array}$$ (2)

The parameter *A* denotes the daily production rate of target cells, which die at rate γ and become infected proportional to the concentration of free viral particles at rate β, assuming random mixing. Infected cells die at rate δ and are cleared of infection at a rate proportional to the size of the immune response and the removal rate α. Free viral particles are produced by infected cells at rate ω and are cleared at rate κ. The immune response grows proportional to the number of infected cells at rate η. This model was fitted to individual plasma viral titers from primary (1°) and secondary (2°) apparent symptomatic (AS) DENV-1 infections [21] using Markov chain Monte Carlo methods [18]. We used *n* = 3,000 random samples from the joint posteriors from that analysis to model viremia trajectories for 1° and 2° AS infections. Inapparent symptomatic (IS) infections are assumed to have similar viremia as AS infections. To adapt this approach to model viremia trajectories of As infections, we relied on the observation by Duong et al. [16] that log_10_ viral titers of As infections were lower on average than those of S infections (76%, CI: 63–88%). To account for uncertainty in this relationship, we took the ratio of 3,000 random samples from the normal distributions of symptomatic (mean: 6.27 +/- SE 0.14 log_10_ cDNA copies/mL) and asymptomatic observed viremia (mean: 4.75 +/- SE 0.39 log_10_ cDNA copies/mL) and reduced each viremia trajectories of AS infections by a random draw from the distribution of fractions to approximate the trajectories of As infections. In doing so, we assumed the duration of viremia to be similar in As and S infections on the basis of limited data from the study performed by Duong et al. [16], which revealed no detectable differences in the duration of viremia between A and S infections.

### Class-specific viremia-infectiousness relationships

To describe the probability of infecting a mosquito given an individual’s viremia (*V*), we used logistic regression models

$$F(V)=\frac{1}{1+e^{-(\beta_{0}+\beta_{1}V)}},$$ (3)

where β_0_ and β_1_ denote the logistic intercept and the slope coefficient for plasma viremia (log_10_ cDNA copies per mL), respectively [49] (see S2 Text for fits to alternative functional forms). We fitted this relationship to data from DENV-infected symptomatic and asymptomatic individuals (S4 Table) and found these relationships to be significantly different across infection classes (i.e., asymptomatic, pre-symptomatic, and post-symptomatic) but not with respect to serotype or pre-exposure history (i.e., primary vs. post-primary infection) [16] (See S2 Text for a formal comparison with other studies on DENV infectiousness [41,42]). Pre-symptomatic individuals become symptomatic after their intrinsic incubation period (IIP) is over, at which time they become subject to a significantly different relationship between viremia and infectiousness (S1 Table). Each of 3,000 samples of symptomatic viremia trajectories had a corresponding duration of the IIP as informed by the posterior distributions derived in [18]. These were paired within a given realization of the regression model with coefficients randomly drawn from the multivariate normal distribution of best fit parameters (S1 Table), so as to explicitly account for uncertainty in these estimates resulting from limited sample sizes in this study, in particular for As infections [16]. The pre-symptomatic parameterization was used before the IIP concludes, and the post-symptomatic parameterization was used afterwards. For the infectiousness of asymptomatic infections, the viremia-infectiousness relationship remained the same over the course of the infection, because the concepts of IIP and onset of symptoms do not apply to asymptomatic infections. To summarize the extent of infectiousness of an individual over the entire course of their infection, we defined net infectiousness as the integral of an infectiousness curve over time

NI=∫F(V(t))dt. (4)

This quantity *NI* is proportional to the expected number of mosquitoes infected by a human infection assuming that biting occurs at a constant rate over the course of the human infection. By extension, the ratio of the net infectiousness of two individuals with two different types of infections is identical to the ratio of the expected number of mosquitoes infected by people with those respective types of infections. Given that we interpret the end of the intrinsic incubation period (IIP) as the beginning of the symptomatic phase of the infection, we also used this distinction to estimate the proportion of infectiousness that occurs prior to symptom onset (NI_PreS_)

$$NI_{PreS}=\frac{\int_{0}^{IIP}F\left(V(t)\right)dt}{NI},$$ (5)

and likewise for the proportion after symptom onset

$$NI_{PostS}=\frac{\int_{IIP}^{\infty }F\left(V(t)\right)dt}{NI}.$$ (6)

### Proportion of 1°, 2°, and post-2° infections as a function of transmission intensity

We calculated the proportion of the population previously exposed to 0 to 4 serotypes as a function of the population’s age distribution and the time-averaged, serotype-specific FoI to which the population is subject. The time-averaged FoI metric (defined below) that we used was assumed to be constant with respect to virus serotype and space. Although DENV FoI is known to exhibit substantial variation with respect to these factors [26,50], we simplified this aspect of our analysis to reflect the average across a wide geographic area or across many realizations of a complex temporal pattern of transmission.

Consistent with these assumptions and with the further assumption of FoI acting as a constant hazard, we represented the proportion pre-exposed to *i* = 0…4 serotypes at age *a* as *e*_*i*_(*a*). After acquiring infection at rate (4-*i*)FoI, an individual has temporary heterologous immunity to all serotypes for a period of average duration σ^-1^. The probability that an individual of age *a* has temporary heterologous immunity after exposure with *i* serotypes is represented by *r*_*i*_ (*a*). Individuals permanently retain immunity to serotypes to which they were previously exposed; i.e., permanent homologous immunity. The dynamics of these classes with respect to age follow

$$\begin{array}{l} \frac{de_{0}}{da}=-4\;\text{FoI}\;e_{0}\\ \frac{dr_{i}}{da}{|}_{i=1\ldots 4}=\left(4-(i-1)\right)\;\text{FoI}\;e_{\left(i-1\right)}-\sigma r_{i}\\ \frac{de_{i}}{da}{|}_{i=1\ldots 4}=\sigma r_{i}-(4-i)\;\text{FoI}\;e_{i}. \end{array}$$ (7)

Accounting for the proportion of the population in each age group *p*(*a*), the population-wide proportion pre-exposed to *i* serotypes is

$$E_{i}=\sum_{a}\left(p(a)e_{i}(a)\right),$$ (8)

where *p*(*a*) was informed by national age distributions from Brazil and Thailand [51], as representative examples of two DENV-endemic regions.

### Proportion of infections of each class among 1°, 2°, and post-2° infections

As+IS:AS ratios for primary and secondary infections were derived from a recent meta-analysis using cohort studies with laboratory-confirmed infections [23]. In brief, a literature search was performed for dengue cohort studies that 1) reported the number of (As+IS) and AS infections and 2) provided lab confirmation of apparent infections [22,52–59] (S3 Table). For a specific time and place *j*, observed infections with pre-exposure to *i* serotypes (*O*_*i*_) were assumed to result from a binomial distribution $O_{i,j}(As+IS)∼Binomial\left(N_{i},\zeta_{i}\lambda_{j}\right),$ where *N*_*i*_ is the number of subjects pre-exposed to *i* serotypes, ζthe (As+IS):AS ratio, and λ the location- and time-specific force of infection. Similarly, the observed AS infections were assumed to result from $O_{i,j}(AS)∼Binomial\left(N_{i},\left(1-\zeta_{i}\right)\lambda_{j}\right).$The models were fit to the data in a Bayesian framework with the R package *RStan* [60]. We assessed observations on post-2° infections [22,28], as collated by [7] in a similar fashion and assumed the number of As+IS infections to have arisen from a binomial distribution $O_{\geq 2}(As+IS)∼Binomial(O_{A+IS}+O_{AS},\xi_{\geq 2}).$ To account for intrinsic variability in the rates, similar models assuming a beta-binomial distribution were assessed and compared to the binomial model using DIC [61]. Given the scarcity of data and the fact that these estimates comprise only a portion of our analysis, we did not estimate λ_i_ as was done in [23]. Had we done so, we would expect our estimates of that quantity to be similar.

To calculate the proportions of infected people who have previously been exposed to zero or one serotype and who experience either an asymptomatic (A) or symptomatic (S) infection, we used *E*_*i*_ and our estimates of As:S ratios for a given pre-exposure history ($\theta_{i}$= 9.2% for 1° and 2° infections, other options are assessed in Fig 6), resulting in

$$\begin{array}{l} \mathrm{Pr}(\text{As})=\sum_{i=1}^{2}E_{i}\theta_{i}\\ \mathrm{Pr}(\text{S})=\sum_{i=1}^{2}E_{i}(1-\theta_{i}). \end{array}$$ (9)

Similarly, the proportion of infections found to be inapparent symptomatic (IS) or apparent symptomatic (AS) follows from the (As+IS):AS ratio, ζ, as

$$\begin{array}{l} \mathrm{Pr}(\text{As+IS})=\sum_{i=1}^{2}\zeta_{i}E_{i}\\ \mathrm{Pr}(\text{AS})=\sum_{i=1}^{2}(1-\zeta_{i})E_{i}\\ \mathrm{Pr}(\text{IS})=\mathrm{Pr}(\text{As+IS})-\mathrm{Pr}(\text{As}). \end{array}$$ (10)

### Theoretical formula for contribution of each infection class to force of infection

Classical epidemiological theory for mosquito-borne pathogen transmission posits that FoI—i.e., the rate at which susceptible individuals become infected—is a function of a number of factors, including infection prevalence among hosts (*X*) and the infectiousness of infected hosts [62]. On a per capita basis, FoI = *bmaY* in Ross-Macdonald models, where *b* is the probability that a susceptible host becomes infected after being bitten by an infectious vector, *m* is the ratio of vectors to hosts, *a* is the daily rate of at which a vector bites, and *Y* is the infection prevalence among vectors. The latter depends further on the daily vector mortality rate *g*, the incubation period *n* in the vector, human infection prevalence *X*, and the probability *c* that a susceptible mosquito becomes infected upon biting an infectious host [62].

To account for the population stratification that is necessary for our analysis, we derived a formula for the FoI that is more generalizable than the classic formula in that it allows for distinct contributions to FoI from different host groups (i.e., As, IS, UAS, and DAS infections with different pre-exposure histories). Specifically, each host group differed in its infectiousness and its overall prevalence in the population. By separating contributions from different host groups to mosquito infectiousness and, in turn, to the FoI on susceptible hosts, we calculated the proportional contribution of people with As infections to FoI by calculating the ratio between the FoI resulting from As infections alone and the total FoI, and similarly for IS, UAS, and DAS infections.

Differentiating between S and As infections, we can describe FoI as

$$\begin{array}{l} {\text{FoI}}_{\text{As}}=bma\frac{ac_{\text{As}}X_{\text{As}}}{g+ac_{\text{As}}X_{\text{As}}}e^{-gn},\\ {\text{FoI}}_{\text{S+As}}=bma\frac{a(c_{\text{As}}+\rho c_{\text{S}})X_{\text{As}}}{g+a(c_{\text{As}}+\rho c_{\text{S}})X_{\text{As}}}e^{-gn}, \end{array}$$ (11)

where ρ is the prevalence of S infections relative to As. The contribution of As infections follows from dividing the quantities in (11) as

$$\frac{{\text{FoI}}_{\text{As}}}{{\text{FoI}}_{\text{S+As}}}=\left(1+\frac{\rho c_{\text{S}}}{c_{\text{As}}}\right)\frac{\frac{g}{a}+c_{\text{As}}X_{\text{As}}}{\frac{g}{a}+(c_{\text{As}}+\rho c_{\text{S}})X_{\text{As}}},$$ (12)

which demonstrates the robustness of our results to different values of *n*, *b*, and *m*.

Under a Ross-Macdonald formulation, the time-invariant infection probability *c* relates to net infectiousness (*NI*) according to NI=c/ι, where $\iota^{-1}$ represents the average duration of the period of infectiousness. The prevalence *X*_*i*_ is proportional to the proportion of the population that has temporary heterologous immunity, *R*_*i*_, according to $X_{i}=R_{i}\sigma /\iota .$The quantity *R*_*i*_ is defined from Eq (7) according to $R_{i}=r_{i}(a)p(a).$ It follows that

$$cX_{i}=NI\sigma R_{i}.$$ (13)

Substituting Eq (13) into Eq (11) to further stratify infection classes and pre-exposure histories gives Eq (1) from the Results section, with *p* = 2 when assessing the impact of 1° and 2° infections on transmission and *p* = 4 when assessing the impact of 1°, 2°, and post-2° infections on transmission. To account for uncertainty in this metric, the relative FoI was calculated for *n* = 3,000 random samples from the distributions of *NI* for each infection class.

### Sensitivity analysis

We performed a sensitivity analysis to assess the impact of uncertainty in As:IS:AS ratios among 1° and 2° infections on the relative contributions to the overall FoI (Fig 6). Additionally, we examined what the contribution of inapparent infections to the FoI would be under the assumption that post-2° infections are similarly infectious as 2° infections and using estimates from the meta-analysis presented in S3 Table to estimate the proportion of post-2° infections to be apparent. This and the assumption we effectively made in our primary analysis—i.e., that post-2° infections make no contribution to transmission—represent two different extremes and therefore provide bounds on the potential sensitivity of our results to alternative assumptions about the contributions to transmission from post-2° infections. Similarly, we addressed uncertainty around the net infectiousness of IS infections by first assuming their net infectiousness to be similar to As infections rather than AS infections, as in the core analysis. We then examined the impact of severe AS infections by adopting fitted dengue hemorrhagic fever (DHF) viremia trajectories [18], DHF rates among S infections of 0.8% and 3% for primary and post-primary infections, respectively [28], and the assumption that all DHF cases are detected. The latter assumption provides an upper bound for the potential contribution of DAS infections for a given detection rate.

### Variance-based sensitivity analysis

To assess the impact of the three main sources of uncertainty in deriving two outcome variables (*Q*), the net infectiousness and the proportion of infectiousness prior to symptoms, we performed a variance-based sensitivity analysis [27]. The variance for both variables was measured under four different scenarios: 1) with all sources of uncertainty and 2–4) with all sources of uncertainty except one. The contribution to the total variance is expressed by the total-effect index

$$T_{i}=1-\frac{Var_{X\sim i}(Q)}{Var(Q)}.$$ (14)

Here, **X** denotes a vector of uncertain model inputs and ~*i* denotes that uncertainty around all inputs was included with the exclusion of *i*. For input *i*, we used the mean of the uncertainty distribution for the quantity of interest. The three main sources of uncertainty are quantities describing temporal patterns of viremia, the relationship between viremia and infectiousness, and the duration of the intrinsic incubation period (IIP).

## Supporting information

S1 Text Assessment of model assumptions and limitations. (PDF)

S2 Text Assessment of infectiousness data. (PDF)

S1 Table Model parameters. (PDF)

S2 Table Probabilistic comparison of net infectiousness uncertainty distributions. (PDF)

S3 Table Cohort studies addressing (As+IS): AS ratios in primary (1°), secondary (2°) infections, and post-2° infections. Studies for 1° and 2° were collated in [23]. Studies used for post-2° [22,28] were derived from [7]. N.S. = not stated.
(XLSX)

S4 Table Data from direct feeding experiment [16] used to inform viremia to infectiousness relationship. (XLSX)

S1 Fig Mean contribution of infection classes to total force of infection (FoI) when accounting for the contribution of post-secondary infections to transmission. The contribution to the total FoI of a class is derived from the ratio between FoI attributable to this class and total FoI, as in Eq (1). The respective net infectiousness is derived from the 3,000 random samples displayed in Fig 3. The infections are further distributed according to the estimated proportion of net infectiousness to occur before and after symptom onset (pre-symptomatic (Eq (5)) and post-symptomatic (hatched lines) (Eq (6)). The histogram shows the distribution of FoI contributions by asymptomatic infections at FoI = 0.1, accounting for parameter uncertainty. Post-secondary infections are assumed to follow the same viremia trajectory as secondary infections. 86% of post-secondary infections are As or IS (S3 Table).
(PDF)

S2 Fig Pre-exposure profile as a function of the force of infection for (a) Brazil, (b) Thailand. The seroprevalence of the population is estimated using a system of ordinary differential equations with state variables denoting the proportion of the population pre-exposed to 0–4 serotypes. Transition to pre-exposure state i occurs at a rate (4-i)FoI. Individuals entering a new pre-exposure state retain temporary heterologous immunity to all serotypes for an average duration of 2 years [64] before becoming susceptible to heterologous serotypes.
(PDF)

S3 Fig Mean contribution of infection classes to total force of infection (FoI) for Thailand. The contribution to the total FoI of a class is derived from the ratio between FoI attributable to this class and total FoI, as in Eq (1). The respective net infectiousness is derived from the 3,000 random samples displayed in Fig 3. The infections are further distributed according to the estimated proportion of net infectiousness to occur before and after symptom onset (pre-symptomatic (Eq (5)) and post-symptomatic (hatched lines) (Eq (6)). The histogram shows the distribution of FoI contributions by asymptomatic infections at FoI = 0.1, accounting for parameter uncertainty.
(PDF)

S4 Fig Variance-based sensitivity analysis. The contribution to the variance represents the total effect index, denoting the contribution of each source of uncertainty to the total variance, including its interactions. (IIP = intrinsic incubation period).
(PDF)

S5 Fig Mean contribution of infection classes to total FoI in an emerging setting. The contribution to the total FoI of a class is derived from the ratio between FoI attributable to this class and total FoI, as in Eq (1). The respective net infectiousness is derived from the 3,000 random samples displayed in Fig 3. The infections are further distributed according to the estimated proportion of net infectiousness to occur before and after symptom onset (pre-symptomatic (Eq (5)) and post-symptomatic (hatched lines) (Eq (6)). The histogram shows the distribution of FoI contributions by asymptomatic infections at FoI = 0.1, accounting for parameter uncertainty.
(PDF)
