# Supplementary material for: Contributions from the silent majority dominate dengue virus transmission
Source: PLoS Pathog. 2018 May 3;14(5):e1006965. doi: 10.1371/journal.ppat.1006965 (PMC5933708; doi:10.1371/journal.ppat.1006965)
Supplement: S1 Text — (PDF) [file ppat.1006965.s001.pdf]

Text S1. Assessment of model assumptions and limitations

Our results support the hypothesis that inapparent infections contribute substantially to DENV transmission. There are a number of uncertainties, however, that underscore the need for future research on the human immune response to DENV infection and correlates of disease severity. Below, we review the main limitations and data gaps identified by our analysis.

- **Other predictors of infectiousness than plasma viral load**. Viremia is typically estimated with the concentration of viral genome copies in plasma. Other factors have been found to influence the probability of transmission to mosquitoes, such as serological response and the day of illness [1]. Similarly, measures of infectious virus across viremia profiles are needed to more fully understand how viremia dynamics relate to a person's infectiousness to mosquitoes.
- **Limited immunological complexity of the within-host model.** In the absence of data on target cell populations or effector immune responses, the ability to fit models with greater complexity and, thereby, to enhance understanding of the human immune response is limited [2,3]. Such studies could help reveal correlates of differences in viremia between primary and secondary infections and gain understanding on the mechanism(s) underlying enhanced infection efficiency in people with asymptomatic and pre-symptomatic infections.
- **Infectiousness prior to onset of symptoms.** The within-host models that we used to estimate viremia in people with symptomatic infections were fitted to post-symptomatic viremia data only. Although realizations of pre-symptomatic viremia were robust to the structural and parameter uncertainty that we explored [4], the

absence of pre-symptomatic data could lead to underestimation of early viremia levels and, as a consequence, underestimation of pre-symptomatic infectiousness. In addition, some parameters that drive early viremia trajectories were pre-assigned due to identifiability restrictions, resulting in underestimation of variation in pre-symptomatic viremia. Obtaining early viremia titer data, either through clustered sampling around index cases (i.e., geographic or contact clusters) or possibly through human challenge studies, depending on the virus strains used, would improve our understanding of early DENV pathogenesis.

- **Viremia trajectories and infectiousness in inapparent symptomatic (IS) infections.** The within-host model was fitted to data of apparent DENV infections (AS). While severe or hospitalized dengue cases have been associated with higher viremia levels than mild AS infections [1,5,6], it is unclear whether this extrapolates to IS infections. Similarly, significant differences between infection efficiency were found between severe and mild AS infections [1] as well as between As and S infections [7], but where IS infections fall on this spectrum remains to be elucidated. Antibodies are believed to play a role in viral clearance and may harbor information on viral trajectories across clinical outcomes [3]. While no significant differences in qualitative and quantitative antibody responses were found in children recovered from a primary IS or AS infection, the breadth in both pre-existing and post-infection antibodies differed significantly between secondary IS and AS infections [8]. Given these uncertainties, we explored the two extreme scenarios: assuming IS infections to be similar to either AS or As infections. The former was treated as the default scenario to ensure consistency with the clinical subgroups used in Duong et al.[7].

The difference between the two scenarios in terms of estimated median contribution of silent infections was 4%.

- **Viremia trajectories in asymptomatic (As) infections.** An empirically supported reduction factor was applied [7] to distinguish between viremia in As and symptomatic (S) infections. However, this factor may be confounded by the timing of the plasma titer measurements [7]. As infections are difficult to identify and the timing of infection is harder to infer than in symptomatic cases. Human challenge studies could aid in clarifying the relationship between viremia progression in relation to clinical outcome [9].
- **Post-secondary infections.** Little is known about the susceptibility to infection, viremia trajectories, and infectiousness of post-secondary infections, in part because determining a person's pre-exposure history after they have been infected with two different DENV serotypes is not reliable [10]. Given the low proportion of AS infections resulting from post-secondary infections (Fig S2), this may well be accompanied with lower viral loads and lower net infectiousness [11,12]. As such, the contribution of inapparent post-secondary infections may be lower than primary and secondary infections. Under the assumption that post-secondary infectiousness is equivalent to that of secondary infections (Fig S1), we estimated that the contribution of As+IS infections could be up to 11% (95% CI 10-13%) higher when accounting for these infections. This should be regarded as an upper bound, because the proportion of As infections among post-secondary infections may well be higher than among primary and secondary infections.

- **Uncertainty and individual heterogeneity.** The steep relationship between viral load and transmission probability [7] in asymptomatic infections is subject to large uncertainty. This results in a broad bimodal pattern in net infectiousness in which a large proportion of asymptomatic infections displays very little infectiousness whereas some are much more infectious than symptomatic individuals. It is not clear how much of this results from parameter uncertainty and how much is a reflection of individual heterogeneity. The fact that the steepness of this relationship is not conserved to the same extent in the data from indirect feeding assays [7] is suggestive of, but not conclusive about, a larger role of uncertainty than individual heterogeneity. Larger sample sizes are required to resolve this issue.
- **Definitions and study designs differ across As:IS:AS rates.** The proportion of apparent infections detected may vary according to the study design used [13], with very active surveillance, as is typical in vaccine trials, resulting in somewhat higher estimates of the proportion of apparent infections [14]. Individuals detected as asymptomatic may become symptomatic later on, something not all study designs account for. This can result in overestimates of As infections at the expense of S infections. A universal, continuous metric for clinical dengue severity could aid in revealing correlates of dengue disease severity that currently go unnoticed in categorical analyses.
- **Additional factors influencing viremia, infectiousness, and clinical outcomes.** While the estimated viral titers used in this analysis were fitted to only DENV-1, these titers may well vary across serotypes [1,2,15], and may be affected by the time since previous infection and the serotype a person was pre-exposed to. Similarly,

infectiousness is found to vary across virus serotypes [1], genotypes [16], and vector-virus genotype interactions [17]. Rates of clinical disease and detection can vary across regions due to factors such as DENV serotype [18], genotype [9,19], the clinical outcome of a previous DENV infection [13,19] and time since a previous outbreak [8], altering the relative contributions of infection classes.

- **Relation between symptoms and detection.** In our analysis, detection rates relied on the assumption that the severity of symptoms is proportional to the proportion of DENV infections detected by disease surveillance systems; i.e., IS are assumed not to be detected. However, health-seeking behavior depends on many factors, not all of which are related to the severity of symptoms. These include socio-economic factors, access to health care, and the perception of the quality of available care, among others [20]. In addition, there can be a delay between symptom onset and health seeking and detection. Therefore, the contribution of individuals prior to detection is almost certainly a conservative underestimate.
- **Extrinsic incubation period (EIP) may vary as a result of viral load** [21-23]. The relatively lower viremia of asymptomatic and secondary infections could increase the length of the incubation period in the mosquito and consequently the net contributions of those infection classes. At a given viremia level, however, people with asymptomatic infections contributed to a higher mosquito viral load than those with symptomatic infections [7]. The impact of lower asymptomatic viremia on the EIP, therefore, may be smaller than expected based solely on viremia. Future xenodiagnostic assessments of infectiousness to mosquitoes would be enhanced by quantifying mosquito infection to test this hypothesis across infection classes.

- **Individuals that develop severe dengue may have a different infectiousness profile**. Viremia estimates from Clapham et al. [4] are consistent with a higher peak viral load and increased cell entry in individuals that develop severe dengue compared to mild dengue cases. It is unclear how infectiousness differs for severe cases, because temporal confounding due to differential health seeking behavior has hampered direct comparison between severe and mild infections [1]. The impact of including severe cases in the analysis is minor due to their small numerical prominence, but their inclusion does increase the contribution of post-symptomatic DAS infections from 1.0% (95% CI: 0.8-1.1%) to 2.1% (95% CI: 0.8-3.6). Severe dengue cases will likely present with impaired mobility and hospitalization, which could also affect their contact rates [24]. However, severe symptoms typically occur after the infectious period has ended, so differences in contact rates between severe and mild dengue cases could end up having a modest impact on their relative contributions to transmission.

SUPPLEMENTARY REFERENCES

1. Nguyen MN, Duong TH, Trung VT, Nguyen TH, Tran CN, Long VT, et al. Host and viral features of human dengue cases shape the population of infected and infectious *Aedes aegypti* mosquitoes. Proc Natl Acad Sci U S A. 2013;110: 9072-9077.

2. Ben-Shachar R, Schmidler S, Koelle K. Drivers of Inter-individual Variation in Dengue Viral Load Dynamics. PLoS Comput Biol. 2016;12: e1005194.

3. Clapham HE, Quyen TH, Kien DTH, Dorigatti I, Simmons CP, Ferguson NM. Modelling Virus and Antibody Dynamics during Dengue Virus Infection Suggests a Role for Antibody in Virus Clearance. PLoS Comput Biol. 2016;12: e1004951.

4. Clapham HE, Tricou V, Van Vinh Chau N, Simmons CP, Ferguson NM. Within-host viral dynamics of dengue serotype 1 infection. J R Soc Interface. 2014;11: 10.1098/rsif.2014.0094.

5. Vaughn DW, Green S, Kalayanarooj S, Innis BL, Nimmannitya S, Suntayakorn S, et al. Dengue viremia titer, antibody response pattern, and virus serotype correlate with disease severity. J Infect Dis. 2000;181: 2-9.

6. Murgue B, Roche C, Chungue E, Deparis X. Prospective study of the duration and magnitude of viraemia in children hospitalised during the 1996-1997 dengue-2 outbreak in French Polynesia. J Med Virol. 2000;60: 432-438.

7. Duong V, Lambrechts L, Paul RE, Ly S, Lay RS, Long KC, et al. Asymptomatic humans transmit dengue virus to mosquitoes. Proc Natl Acad Sci U S A. 2015;112: 14688-14693.

8. Corbett KS, Katzelnick L, Tissera H, Amerasinghe A, de Silva AD, de Silva AM. Preexisting neutralizing antibody responses distinguish clinically inapparent and apparent dengue virus infections in a Sri Lankan pediatric cohort. J Infect Dis. 2015;211: 590-599.

9. Mammen M, Lyons A, Innis B, Sun W, McKinney D, Chung R, et al. Evaluation of dengue virus strains for human challenge studies. Vaccine. 2014;32: 1488-1494.

10. Guzmán MG, Kourí G. Dengue diagnosis, advances and challenges. Int J Infect Dis. 2004;8: 69-80.

11. Olkowski S, Forshey BM, Morrison AC, Rocha C, Vilcarromero S, Halsey ES, et al. Reduced risk of disease during postsecondary dengue virus infections. J Infect Dis. 2013;208: 1026-1033.

12. Montoya M, Gresh L, Mercado JC, Williams KL, Vargas MJ, Gutierrez G, et al. Symptomatic versus inapparent outcome in repeat dengue virus infections is influenced by the time interval between infections and study year. PLoS Negl Trop Dis. 2013;7: e2357.

13. Clapham HE, Cummings DA, Johansson MA. Immune status alters the probability of apparent illness due to dengue virus infection: Evidence from a pooled analysis across multiple cohort and cluster studies. PLoS Negl Trop Dis. 2017;11: e0005926.

14. Ferguson NM, Rodríguez-Barraquer I, Dorigatti I, Mier-y-Teran-Romero L, Laydon DJ, Cummings DA. Benefits and risks of the Sanofi-Pasteur dengue vaccine: Modeling optimal deployment. Science. 2016;353: 1033-1036.

15. Ferguson NM, Kien DT, Clapham H, Aguas R, Trung VT, Chau TN, et al. Modeling the impact on virus transmission of Wolbachia-mediated blocking of dengue virus infection of *Aedes aegypti*. Sci Transl Med. 2015;7: 279ra37.

16. Lambrechts L, Fansiri T, Pongsiri A, Thaisomboonsuk B, Klungthong C, Richardson JH, et al. Dengue-1 virus clade replacement in Thailand associated with enhanced mosquito transmission. J Virol. 2012;86: 1853-1861.

17. Lambrechts L, Knox TB, Wong J, Liebman KA, Albright RG, Stoddard ST. Shifting priorities in vector biology to improve control of vector-borne disease. Trop Med Int Health. 2009;14: 1505-1514.

18. Grange L, Simon-Loriere E, Sakuntabhai A, Gresh L, Paul R, Harris E. Epidemiological risk factors associated with high global frequency of inapparent dengue virus infections. Frontiers in immunology. 2014;5.

19. Katzelnick LC, Montoya M, Gresh L, Balmaseda A, Harris E. Neutralizing antibody titers against dengue virus correlate with protection from symptomatic infection in a longitudinal cohort. Proc Natl Acad Sci U S A. 2016;113: 728-733.

20. Khun S, Manderson L. Health seeking and access to care for children with suspected dengue in Cambodia: an ethnographic study. BMC Public Health. 2007;7: 1.

21. Watts DM, Burke DS, Harrison BA, Whitmire RE, Nisalak A. Effect of temperature on the vector efficiency of Aedes aegypti for dengue 2 virus. Am J Trop Med Hyg. 1987;36: 143-152.

22. Fontaine A, Jiolle D, Moltini-Conclois I, Lequime S, Lambrechts L. Excretion of dengue virus RNA by *Aedes aegypti* allows non-destructive monitoring of viral dissemination in individual mosquitoes. Sci Rep. 2016;6: 24885.

23. Bates M, Roca-Garcia M. Laboratory Studies of the Saimiri-Haemagog'us Cycle of Jungle Yellow Fever. American Journal of Tropical Medicine. 1945;25: 203-216.

24. Perkins TA, Paz-Soldan VA, Stoddard ST, Morrison AC, Forshey BM, Long KC, et al. Calling in sick: impacts of fever on intra-urban human mobility. Proc Biol Sci. 2016;283: 10.1098/rspb.2016.0390.
