# Supplementary material for: Contributions from the silent majority dominate dengue virus transmission
Source: PLoS Pathog. 2018 May 3;14(5):e1006965. doi: 10.1371/journal.ppat.1006965 (PMC5933708; doi:10.1371/journal.ppat.1006965)
Supplement: S2 Text — (PDF) [file ppat.1006965.s002.pdf]

Text S2. Assessment of infectiousness data.

Generalizability across infectiousness data sets

To explore the generalizability of the results as presented in Fig 2, we fit the logistic model defined in equation (3) of the main text separately to data from direct feeding experiments on people with symptomatic infections reported from three different studies [1-3] (see osf.io/pjbhz for data and code). In addition, we fit the logistic model to data from these three studies combined (Figure S2.1). The best-fit curves associated with the three data sets were significantly different from another (likelihood ratio test: df = 4, p-value = 1.19e-23). Pairwise comparisons between the data sets confirmed significant differences between any two dose-response curves, even though the fits to the Duong et al. data set and the Nguyen et al. data set are qualitatively similar (Figure S2.1) (p-values Duong vs Ferguson: 8.8e-6, Duong vs Nguyen: 7.5e-19, and Ferguson vs Nguyen: 8.9e-12).

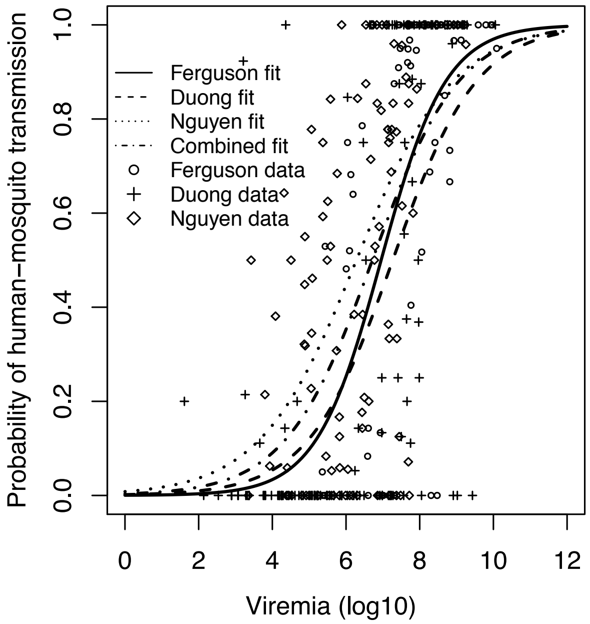

Fig S2.1. Logistic curves fitted to data sets on direct feeding on symptomatic DENV-infected individuals.

Table S2.1. Dose-response curves for three datasets with distinct functional forms.

Data set	Functional form	β_0 (95% CI)	p	β_1 (95% CI)	p	LL	AIC	dAIC
**Duong et al.**	Logistic	-6.37 (-7.12, -5.62)	0	0.88 (0.77, 0.98)	0	927	**931**	0
	Hill	264.61 (158.68, 370.54)	$9.78x10^{-4}$	2.79 (2.58, 3.01)	0	1031	1036	105
	Ferguson	7.95 (7.74, 8.15)	0	4.07 (3.58, 4.56)	0	954	959	28
**Ferguson et al.**	Logistic	-7.91 (-8.82, -6.98)	0	1.14 (1.01, 1.26)	0	1043	1047	6
	Hill	763.22 (423.60, 1102.85)	$1.06x10^{-5}$	3.66 (3.44, 3.89)	0	1178	1183	142
	Ferguson	7.50 (7.39, 7.61)	0	5.17 (4.63, 5.71)	0	1037	**1041**	0
**Nguyen et al.**	Logistic	-4.79 (-5.40, -4.19)	0	0.76 (0.67, 0.86)	0	933	**937**	0
	Hill	495.56 (143.97, 847.15)	$5.75x1^{-3}$	3.42 (3.03, 3.81)	0	969	973	46
	Ferguson	6.97 (6.82, 7.13)	0	3.44 (3.02, 3.86)	0	933	**937**	0

Assessment of functional forms

To explore the appropriateness of a logistic model to describe the dose-response curves, we fitted three different functional forms to the data sets: the logistic model (eqn. 2 in main text), the Hill function, $F(V) = V^{\beta_0}/\beta_1 + V^{\beta_0}$, and the model used by [2], which we will refer to as the Ferguson model, $F(V) = 1 - \exp\,(\frac{-V}{\beta_0} + V)^{\beta_1}$. Goodness of fit of each model variant was assessed using AIC (Table S2.1). The data from [1] were best described by the logistic model, whereas the data from [2] and [3] were described equally well by the logistic model and the Ferguson model. Model fits between the logistic model and the Ferguson model are not qualitatively different (Fig S2.2) and have differences in AIC values of 0 and 6 in two cases. A difference of 5 is frequently interpreted as lack of a significant difference in model fit [4].

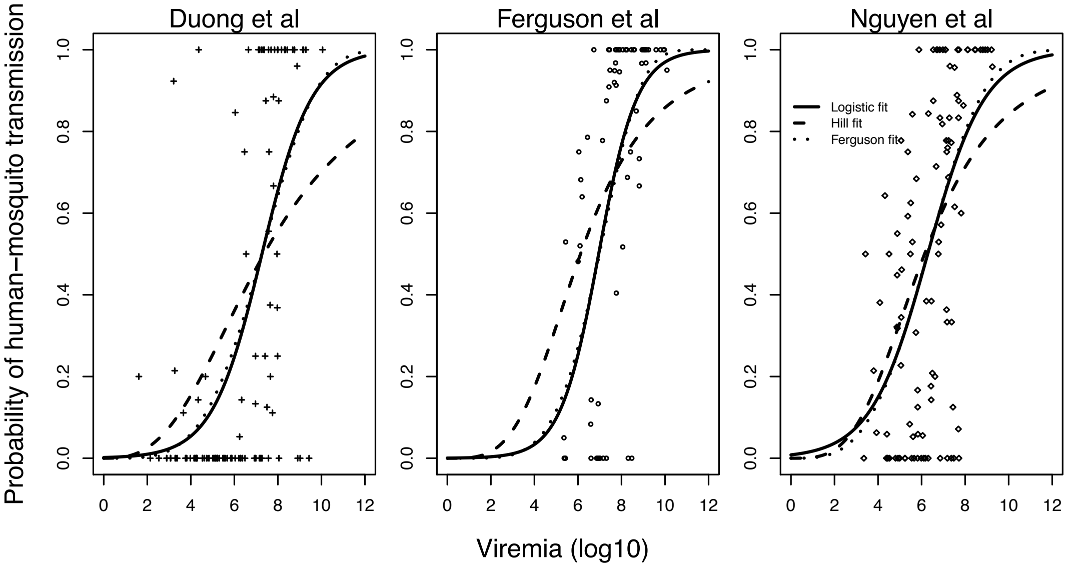

Fig S2.2. Comparison between three different functional forms fitted to data sets from three different studies.

Differences in infectiousness across serotypes

Both [2] and [3] reported significantly different dose-response relationships among serotypes in symptomatic individuals. To assess the extent to which this difference can be explained by infection class, we re-evaluated the difference in dose-response curves between serotypes both for the symptomatic subset and for the full dataset from [1] using logistic regression. Fitting the logistic model to the symptomatic subset supports the findings of the other studies that the dose-response curves vary significantly across serotypes. Specifically, DENV-1 was associated with a curve significantly different from that of DENV-2 ($p = 0.005$), whereas DENV-4 was not ($p = 0.44$). Notably, results about which serotype exhibits the most efficient infectiousness to mosquitoes are mostly consistent across studies, with DENV-1 transmitting most and DENV-3 least efficiently (Fig S2.3 and Table S2.4).

The effect of the different serotypes on dose-response curves changes when assessing the full Duong et al. data set and accounting for infection class. As described by Duong et al., the minimal adequate model does not include serotypes (Table 3) (serotype dropped from model based on likelihood ratio test, $p = 0.54$). There was a significant effect of serotype in the indirect-feeding cohort, indicating that there may have been differences between the cohorts of individuals based around each feeding method.

Table S2.3. Minimal adequate model logistic regression on Duong et al. data set

Factor	Coefficient	SE	p
**Intercept**	-6.36	0.33	<2e-16
**Log_{10}(viremia)**	0.82	0.05	<2e-16
**Infection class (asymptomatic)**	1.43	0.35	3.75e-5
**Infection class (presymptomatic)**	1.63	0.17	<2e-16
**Sex**	0.66	0.13	3.56e-7

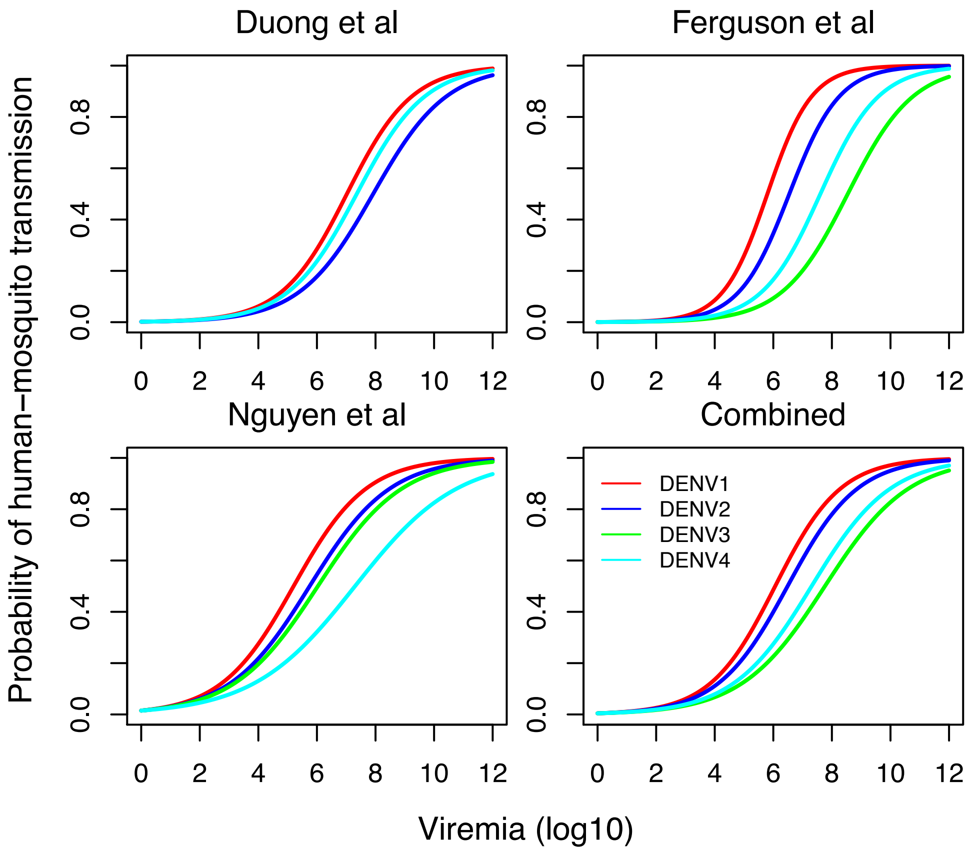

Fig S2.3. Comparative analysis of serotype-specific model fits to three different data sets on symptomatic cases only (logistic model).

Table S2.4. Ranking of most infectious serotypes by study, in decreasing order of infectiousness to mosquitoes per viremic particle in plasma.

Data set	Rank 1	Rank 2	Rank 3	Rank 4
**Duong et al**	DENV 1	DENV 4	DENV 2	NA
**Ferguson et al**	DENV 1	DENV 2	DENV 4	DENV 3
**Nguyen et al**	DENV 1	DENV 2	DENV 3	DENV 4
**Combined**	DENV 1	DENV 2	DENV 4	DENV 3

The role of overdispersal

We explored whether the infection data were over-dispersed using beta-binomial logistic regression, which effectively allows for heterogeneity in dose-response relationships among individuals from which the data points were derived. Based on AIC of models with or without overdispersal (including viremia and infection class), we found no support for accounting for overdispersal in the model (AIC for binomial model: 135; AIC for beta-binomial model: 139). As to be expected, accounting for overdispersal resulted in wider confidence intervals (Fig S2.4).

Table S2.5. Minimal adequate model logistic regression on Duong et al. data set accounting for overdispersal.

Factor	Coefficient	SE	p
**Intercept**	-4.28	0.49	3.5e-15
**$\log_{10}$(viremia)**	0.55	0.07	1.4e-13
**Infection class (asymptomatic)**	0.75	0.85	0.38
**Infection class (presymptomatic)**	1.52	0.36	3.54e-5

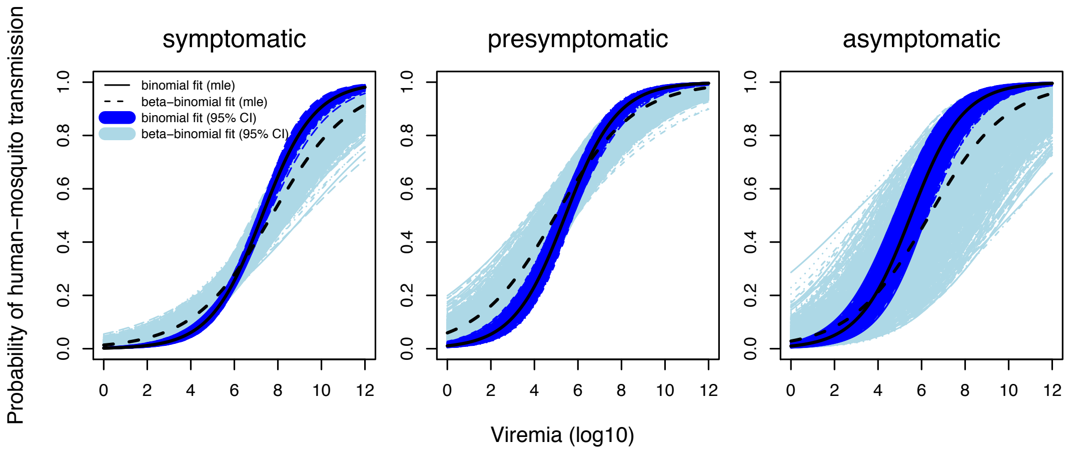

Fig S2.4. Comparative analysis of logistic model fits to Duong et al. data assuming overdispersal of infection data (light blue) or not (dark blue). Different lines represent unique draws from the posterior distribution of parameter values.

Accounting for infection class specific slopes

Lastly, we assessed whether there is statistical support for the possibility that the slopes of the logistic dose-response curves vary among infection classes. A likelihood ratio test between a model with and without infection class-specific slopes showed statistical support for the former ($p = 0.0003$, $df = 2$). This indicates that there is a distinct dose-response curve for asymptomatic and pre-symptomatic infections (Fig S2.6), whereas curves for asymptomatic and pre-symptomatic infections were estimated to be similar when fitted using the simpler model variant (Fig S2.5).

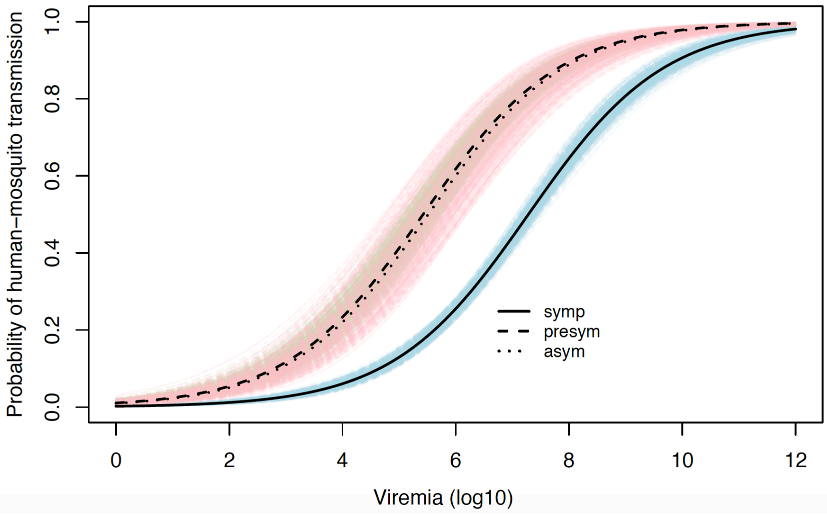

Fig S2.5. Logistic model fits to Duong et al. data by infection class with 95% credible intervals (shaded).

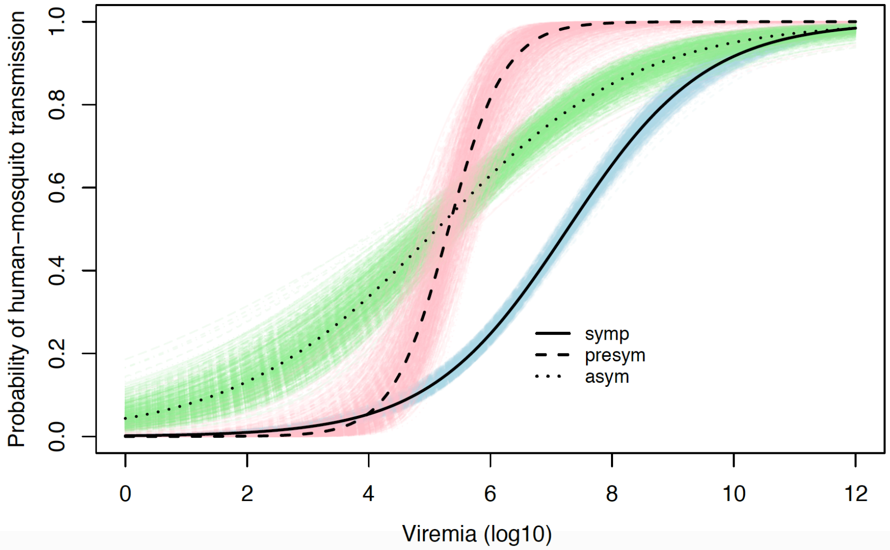

Fig S2.6. Logistic model fits to Duong et al. data by infection class with 95% credible intervals (shaded), allowing for random intercepts.

SUPPLEMENTARY REFERENCES

1. Duong V, Lambrechts L, Paul RE, Ly S, Lay RS, Long KC, et al. Asymptomatic humans transmit dengue virus to mosquitoes. Proc Natl Acad Sci U S A. 2015;112: 14688-14693.

2. Ferguson NM, Kien DT, Clapham H, Aguas R, Trung VT, Chau TN, et al. Modeling the impact on virus transmission of Wolbachia-mediated blocking of dengue virus infection of *Aedes aegypti*. Sci Transl Med. 2015;7: 279ra37.

3. Nguyen MN, Duong TH, Trung VT, Nguyen TH, Tran CN, Long VT, et al. Host and viral features of human dengue cases shape the population of infected and infectious *Aedes aegypti* mosquitoes. Proc Natl Acad Sci U S A. 2013;110: 9072-9077.

4. Burnham KP, Anderson DR. Model selection and multimodel inference: a practical information-theoretic approach. : Springer Science & Business Media; 2003.
