# Supplementary material for: Contributions from the silent majority dominate dengue virus transmission
Source: PLoS Pathog. 2018 May 3;14(5):e1006965. doi: 10.1371/journal.ppat.1006965 (PMC5933708; doi:10.1371/journal.ppat.1006965)
Supplement: S1 Table — (PDF) [file ppat.1006965.s003.pdf]

Table S1. Model parameters.

	Parameter	Symbol	Value (95% CI)	Equation	Ref.
**Modeling individual viremia trajectories**	Uninfected target cells	x	A/γ (initial)	2	[1]
	Infected target cells	y	0 (initial)	2	[2]
	Free viral particles	v	1 (initial)	2	[2]
	Clearing immune response	z	1°S: 0.347 (0.285,0.548)<br>2°S: 0.411(0.248,0.594)<br>2°DHF: 0.380 (0.0298,0.526) (initial)	2	[2]
	Daily production of target cells	A	$1.4\text{x}10^{6}$	2	[3]
	Death rate target cells	γ	0.14	2	[1]
	Infection rate	β	1°S: 1.72 (1.50,1.98)<br>2°S: 2.30 (2.14,2.50)<br>2°DHF: 2.82 (2.58,3.23) ($\text{x}10^{-10}$)	2	[2]
	Death rate infected target cells	δ	0.14	2	[1]
	Removal rate infected cells	α	0.001	2	[2]
	Viral particle production rate	ω	$1 \text{ x } 10^{4}$	2	[4]
	Viral particle clearance rate	κ	1°S: 3.48 ()<br>2°S: 5.29 ()<br>2°DHF: 6.07 ()	2	[2]
	Growth rate immune response	η	1°S: $1.29\text{x}10^{-5}$ ($7.96 \text{ x } 10^{-7}$, $1.09\text{x}10^{-3}$)<br>2°S: $2.95\text{x}10^{-5}$ ($7.36 \text{ x } 10^{-7}$, $3.95\text{x}10^{-3}$)<br>2°DHF: $2.71\text{x}10^{-6}$ ($5.01\text{x}10^{-7}$, 0.224)	2	[2]
**Infectiousness calculations**	Viremia ($\log_{10}$ cDNA copies/mL)	V	-	3-6	
	Logistic intercept	β_0	A: -11.50 (-17.59,-4.16)<br>Pre-sym S: -3.09 (-4.24,-2.01)<br>Post-sym S: -6.37 (-7.12, -5.62)	3	[5]
	Slope coefficient	β_1	A: 2.16 (0.92, 3.42)<br>Pre-sym S: 0.60 (0.43,0.77)<br>Post-sym S: 0.88 (0.77, 0.98)	3	[5]
**Population seroprofile calculation**	Age	a		7-8	
	Proportion pre-exposed to i serotypes at age a	$e_i(a)$		7-8	
	Proportion cross-immune after exposure with i serotypes at age a	$r_i(a)$		7	
	Duration cross-immune period	σ^{--1}	2 years	1,7,13	[6]
	Proportion of the population in each age group	$p(a)$		8	
	Proportion of population pre-exposed to i serotypes	E_i		8-10	
	Proportion of population cross-immune after pre-	R_i		1,13	

	exposure to *i* serotypes				
**Meta-analysis of (A+IS):AS ratios**					
**Disease outcome calculation**	Asymptomatic to symptomatic ratio given pre-exposure to *i* serotypes	θ_i	9.2%	9	[5]
	Inapparent to apparent ratio given pre-exposure to *i* serotypes	ξ_i	1°: 0.82 (0.81,0.84)<br>2°: 0.76 (0.74,0.78)<br>>2°:0.86 (0.63,0.97)	10	[7]
	Location and time-specific force of infection	λ_j			
**Contribution of each infection class to force of infection**	Infection prevalence hosts	X		11-13	
	Probability that a susceptible host becomes infected upon a bite of an infectious vector	b	Cancels out	1,11	
	Ratio of vectors to hosts	m	Cancels out	1,11	
	Bites per vector lifetime	a/g	4	1,11-12	
	Infection prevalence vectors	Y			
	Extrinsic incubation period	n	Cancels out	1,11	
	Probability that a susceptible vector becomes infected upon biting an infectious host given infectious class A or S	$c_{A\ or\ S}$		11-13	
	Infectious period	ι^{-1}	5		

References

1. Gonzalez-Mejia ME, Doseff AI. Regulation of monocytes and macrophages cell fate. Front Biosci. 2009;14: 2413-2431.

2. Clapham HE, Tricou V, Van Vinh Chau N, Simmons CP, Ferguson NM. Within-host viral dynamics of dengue serotype 1 infection. J R Soc Interface. 2014;11: 10.1098/rsif.2014.0094.

3. iGPnotebook. White cell count. http://www.gpnotebook.co.uk (accessed 10 October 2013)

4. Cologna R, Rico-Hesse R. American genotype structures decrease dengue virus output from human monocytes and dendritic cells. J Virol. 2003;77: 3929-3938.

5. Duong V, Lambrechts L, Paul RE, Ly S, Lay RS, Long KC, et al. Asymptomatic humans transmit dengue virus to mosquitoes. Proc Natl Acad Sci U S A. 2015;112: 14688-14693.

6. Reich NG, Shrestha S, King AA, Rohani P, Lessler J, Kalayanarooj S, et al. Interactions between serotypes of dengue highlight epidemiological impact of cross-immunity. J R Soc Interface. 2013;10: 20130414.

7. Clapham HE, Cummings DA, Johansson MA. Immune status alters the probability of apparent illness due to dengue virus infection: Evidence from a pooled analysis across multiple cohort and cluster studies. PLoS Negl Trop Dis. 2017;11: e0005926.
