# Supplementary material for: Contributions from the silent majority dominate dengue virus transmission
Source: PLoS Pathog. 2018 May 3;14(5):e1006965. doi: 10.1371/journal.ppat.1006965 (PMC5933708; doi:10.1371/journal.ppat.1006965)
Supplement: S2 Table — (PDF) [file ppat.1006965.s004.pdf]

Table S2. Probabilistic comparison of net infectiousness uncertainty distributions.

		Asymptomatic		Symptomatic	
		Primary	Secondary	Primary	Secondary
Asymptomatic	Primary	0.50	0.44	0.55	0.50
	Secondary	0.56	0.50	0.64	0.58
Symptomatic	Primary	0.45	0.36	0.50	0.38
	Secondary	0.50	0.42	0.62	0.50

Probability that a randomly selected value of net infectiousness from the uncertainty distribution for individuals of the type specified by the column is greater than a randomly selected value for individuals of the type specified by the row. For example, there is a 57% chance that asymptomatic primary infections have a higher net infectiousness than asymptomatic secondary infections. To provide context, we note that this probability is 0.50 for identical distributions, <0.50 when the random variable on the column is smaller than the one on the row (blue), and >0.50 when the random variable on the column is larger than the one on the row (red).
