# Supplementary figures and images for: Contributions from the silent majority dominate dengue virus transmission
Source: PLoS Pathog. 2018 May 3;14(5):e1006965. doi: 10.1371/journal.ppat.1006965 (PMC5933708; doi:10.1371/journal.ppat.1006965)

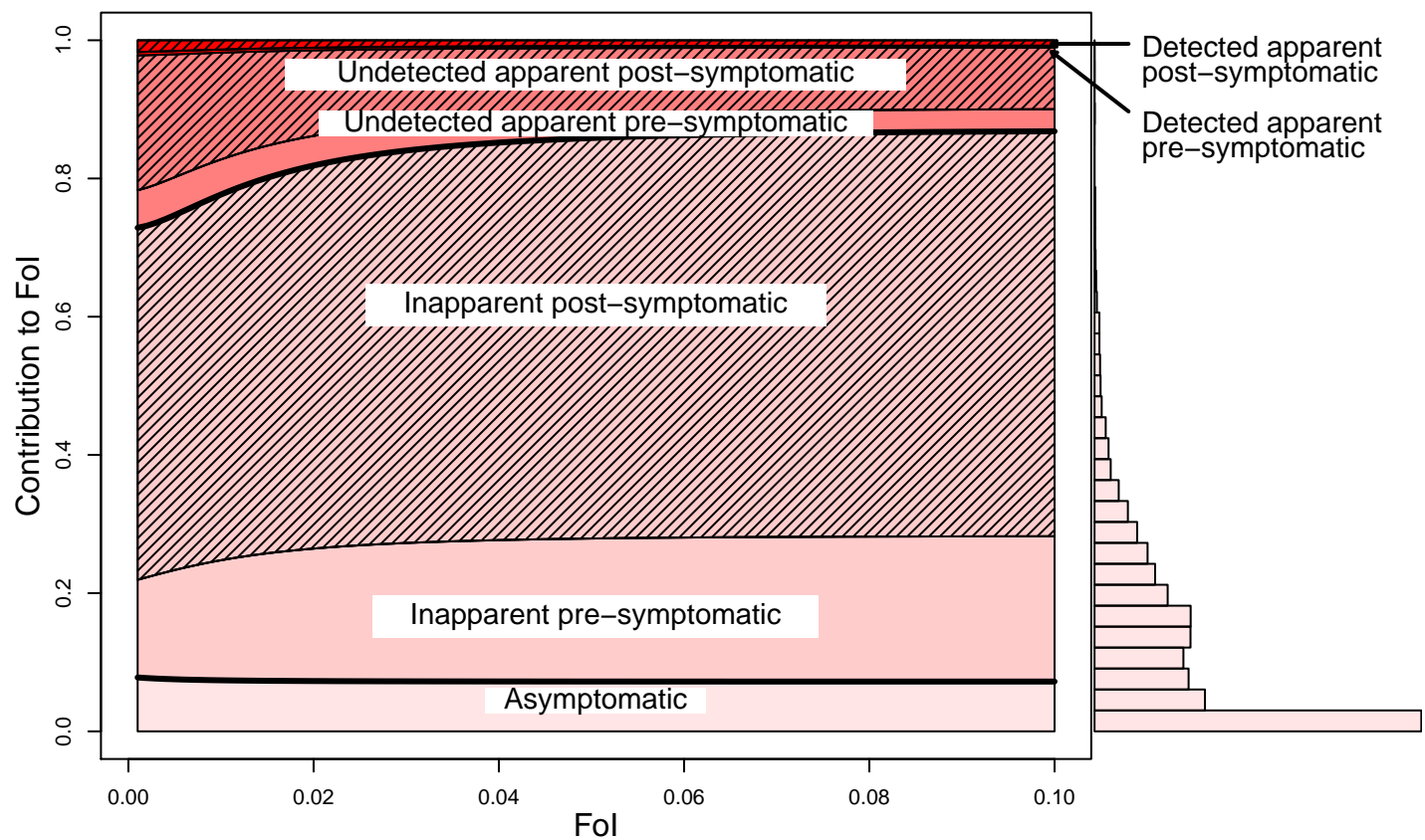

Supplement: S1 Fig — The contribution to the total FoI of a class is derived from the ratio between FoI attributable to this class and total FoI, as in Eq (1). The respective net infectiousness is derived from the 3,000 random samples displayed in Fig 3. The infections are further distributed according to the estimated proportion of net infectiousness to occur before and after symptom onset (pre-symptomatic (Eq (5)) and post-symptomatic (hatched lines) (Eq (6)). The histogram shows the distribution of FoI contributions by asymptomatic infections at FoI = 0.1, accounting for parameter uncertainty. Post-secondary infections are assumed to follow the same viremia trajectory as secondary infections. 86% of post-secondary infections are As or IS (S3 Table). (PDF) [file ppat.1006965.s007.pdf]

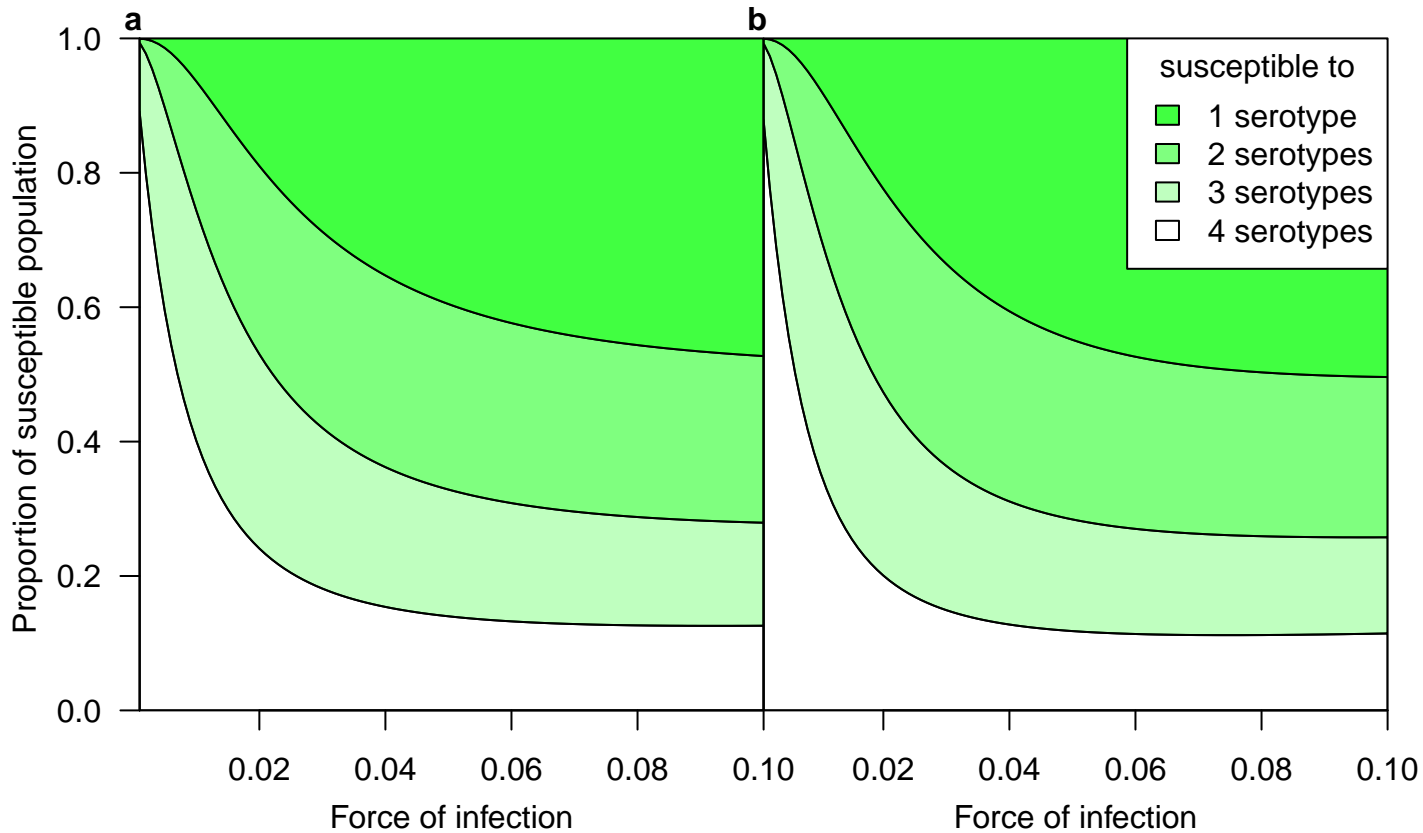

Supplement: S2 Fig — The seroprevalence of the population is estimated using a system of ordinary differential equations with state variables denoting the proportion of the population pre-exposed to 0–4 serotypes. Transition to pre-exposure state i occurs at a rate (4-i)FoI. Individuals entering a new pre-exposure state retain temporary heterologous immunity to all serotypes for an average duration of 2 years [64] before becoming susceptible to heterologous serotypes. (PDF) [file ppat.1006965.s008.pdf]

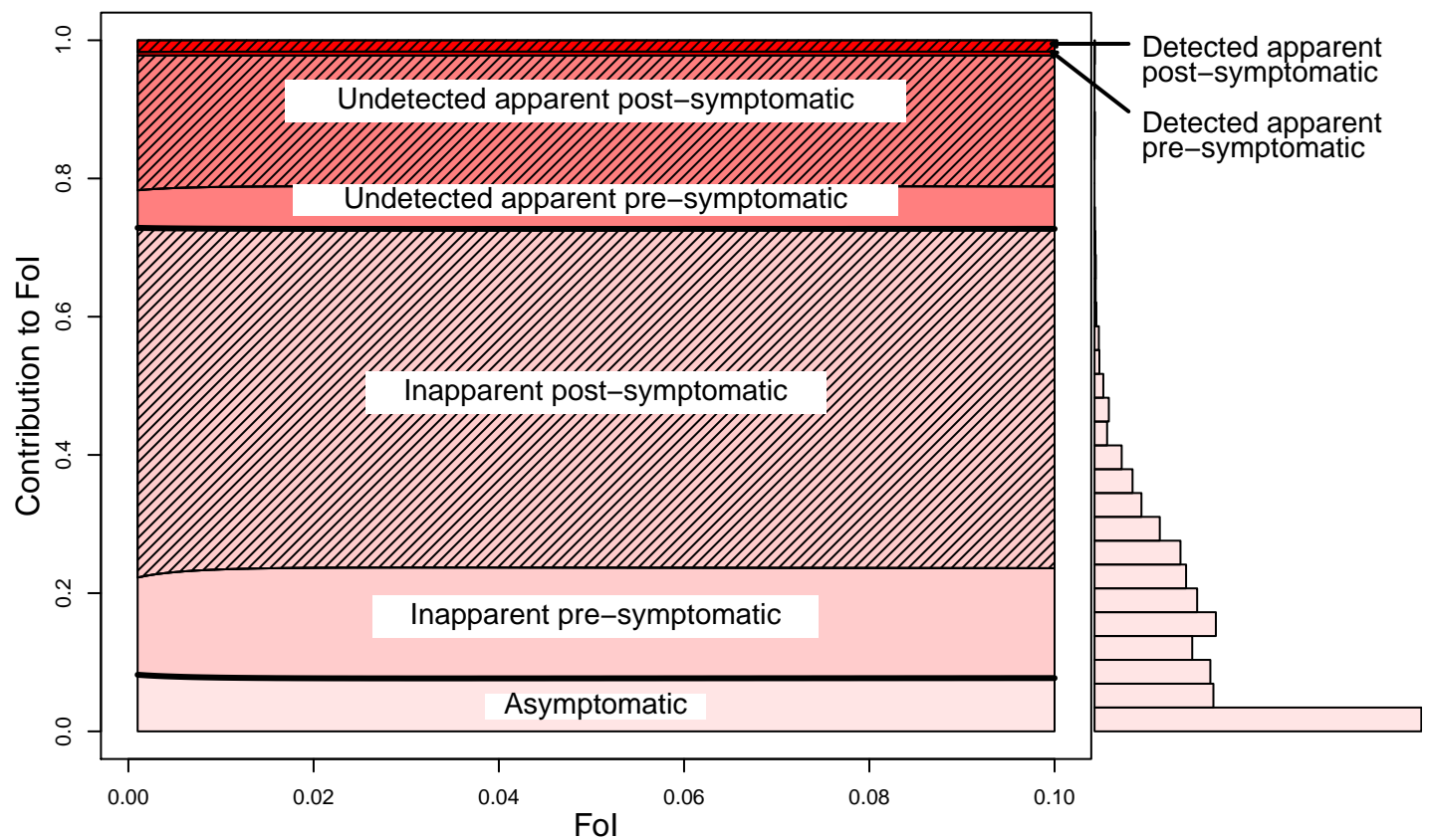

Supplement: S3 Fig — The contribution to the total FoI of a class is derived from the ratio between FoI attributable to this class and total FoI, as in Eq (1). The respective net infectiousness is derived from the 3,000 random samples displayed in Fig 3. The infections are further distributed according to the estimated proportion of net infectiousness to occur before and after symptom onset (pre-symptomatic (Eq (5)) and post-symptomatic (hatched lines) (Eq (6)). The histogram shows the distribution of FoI contributions by asymptomatic infections at FoI = 0.1, accounting for parameter uncertainty. (PDF) [file ppat.1006965.s009.pdf]

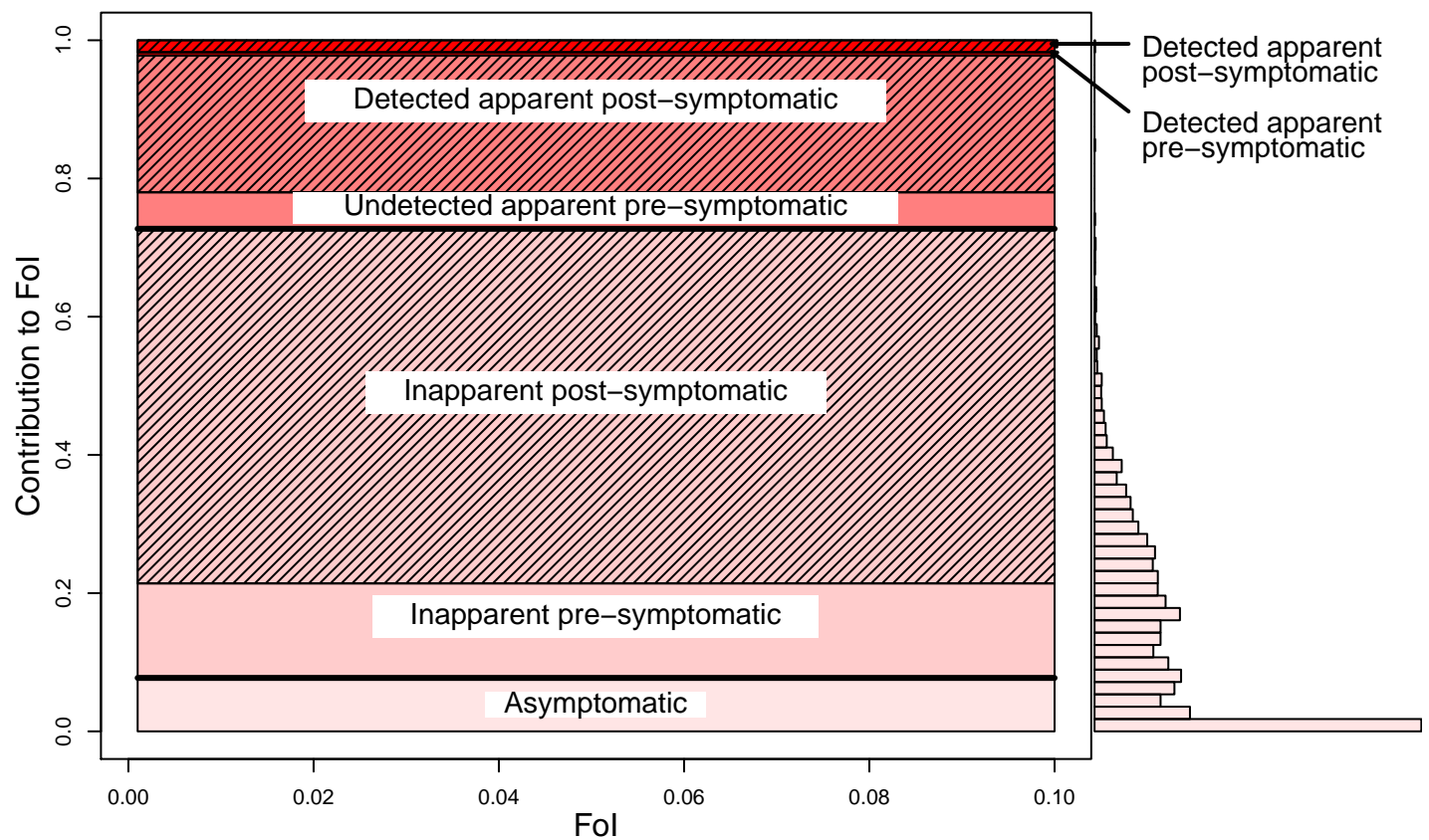

Supplement: S5 Fig — The contribution to the total FoI of a class is derived from the ratio between FoI attributable to this class and total FoI, as in Eq (1). The respective net infectiousness is derived from the 3,000 random samples displayed in Fig 3. The infections are further distributed according to the estimated proportion of net infectiousness to occur before and after symptom onset (pre-symptomatic (Eq (5)) and post-symptomatic (hatched lines) (Eq (6)). The histogram shows the distribution of FoI contributions by asymptomatic infections at FoI = 0.1, accounting for parameter uncertainty. (PDF) [file ppat.1006965.s011.pdf]
