# Supplementary material for: Contributions from the silent majority dominate dengue virus transmission
Source: PLoS Pathog. 2018 May 3;14(5):e1006965. doi: 10.1371/journal.ppat.1006965 (PMC5933708; doi:10.1371/journal.ppat.1006965)
Supplement: S4 Fig — The contribution to the variance represents the total effect index, denoting the contribution of each source of uncertainty to the total variance, including its interactions. (IIP = intrinsic incubation period). (PDF) [file ppat.1006965.s010.pdf]

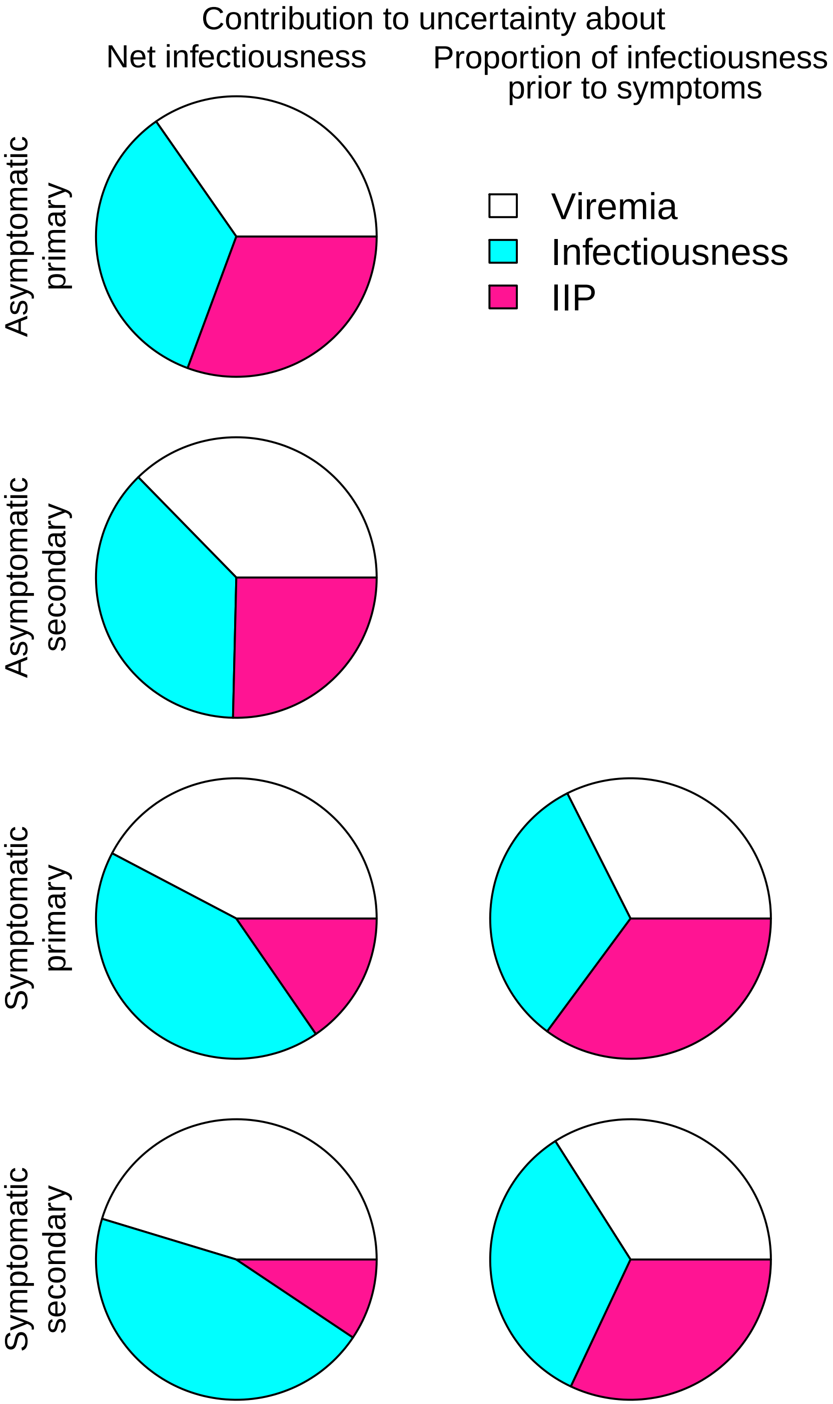
Contribution to uncertainty about
Net infectiousness
Proportion of infectiousness prior to symptoms
Asymptomatic primary
Asymptomatic secondary
Symptomatic primary
Symptomatic secondary
Viremia
Infectiousness
IIP
